# The Effects of Advanced Resistance Training Prescription Methods on Strength, Power, Hypertrophy, and Performance Adaptations in Healthy Adults: A Systematic Review and Bayesian Network Meta-analysis

**DOI:** 10.1007/s40279-026-02428-1

**Published:** 2026-04-08

**Authors:** Nicholas Cowley, Vaughan Nicholson, Ryan Timmins, Gabriella Munteanu, Jonathon Weakley

**Affiliations:** 1 School of Behavioural and Health Sciences, Australian Catholic University, McAuley at Banyo, Brisbane, QLD Australia; 2 Sports Performance, Recovery, Injury and New Technologies (SPRINT) Research Centre, Australian Catholic University, Brisbane, QLD Australia; 3 School of Allied Health, Australian Catholic University, Brisbane, QLD Australia; 4 Carnegie Applied Rugby Research (CARR) Centre, Carnegie School of Sport, Leeds Beckett University, Leeds, UK

## Abstract

**Background:**

Advanced resistance training methods are commonly promoted as superior for long-term improvements in physical qualities and performance capacities. However, at present, there is no clear evidence that advanced resistance training methods are better than traditional approaches, or than one another, in promoting adaptation in healthy adults.

**Objectives:**

This systematic review and Bayesian network meta-analysis aimed to (1) compare advanced methods of resistance training prescription and their effects on strength, power, hypertrophy, and performance adaptations in healthy adults; (2) identify variables that may influence adaptations following specific resistance training methods; and (3) provide a rank order of advanced resistance training methods in their effectiveness for developing each physical capacity.

**Methods:**

This review was conducted using the Preferred Reporting Items for Systematic Reviews and Meta-Analyses extension statement for network meta-analyses (PRISMA-NMA). Five databases were searched, with studies included if they were peer-reviewed investigations, written in English, and compared at least two of eight resistance training methods (i.e. traditional resistance training or one of seven advanced methods). Furthermore, studies must have assessed strength, power, hypertrophy, jump, or sprint adaptations. Risk of bias was assessed using the Cochrane Risk of Bias tool V.2. Bayesian network meta-analyses and meta-regressions were performed to quantify the differences between resistance training methods and identify any variables that may moderate adaptations.

**Results:**

Strength and power adaptations were similar across all resistance training methods, with all relative effects from Bayesian network meta-analyses having 95% credible intervals (CrIs) that crossed zero. Consequently, rankings of resistance training methods for strength and power adaptations should be interpreted cautiously due to the lack of any meaningful differences across the separate networks—although network meta-regressions revealed that rest redistribution schemes may be more beneficial for the development of strength in females in comparison to males. Flywheel training resulted in superior jump adaptations, with a greater benefit seen from shorter interventions and lower training volume load. However, rest redistribution schemes resulted in greater jump adaptations than flywheel training with a higher frequency of sessions. A systematic review of the literature also revealed no resistance training method that consistently induces superior adaptations for hypertrophy and sprint performance.

**Conclusions:**

When aiming to develop a range of physical capacities, there is no clear benefit from using advanced methods over traditional resistance training for inducing adaptation in untrained to moderately trained individuals. However, advanced methods can still be beneficial for practitioners to implement. If practitioners prescribe advanced resistance training methods, it is important for them to consider individual athlete needs, the training cycle, and other training variables that may affect short-term responses as well as chronic adaptations. Future research should target the limitations of the current literature and recruit participants with a greater training age and relative strength, across a range of outcome measures.

**Supplementary Information:**

The online version contains supplementary material available at 10.1007/s40279-026-02428-1.

## Key Points

**Table:** 

Bayesian network meta-analyses revealed no meaningful difference (i.e. 95% credible interval [CrI] crossed zero) when comparing the strength and power adaptations of advanced and traditional resistance training methods in healthy adults. However, rest redistribution schemes may be beneficial for developing strength in females in comparison to males. Flywheel training was superior for developing jump performance, particularly with shorter intervention durations, lower training volume loads, and less frequent training sessions.
Through systematic review of the literature, no method of resistance training showed consistently superior hypertrophy and sprint performance adaptations in healthy adults.
Most research investigating adaptations following advanced resistance training methods has used relatively untrained populations. Therefore, further high-quality research is needed with trained individuals (e.g. > 5 years resistance training experience or a relative measure of strength such as ≥ 2 × bodyweight back squat) across a comprehensive range of outcome measures (i.e. strength, power, hypertrophy, jump performance, sprint performance).

## Introduction

Resistance training can improve a range of physical qualities (e.g. strength, power, muscle mass) and performance outcomes (e.g. jumping and sprinting) [1–3]. The prescription of resistance training involves the manipulation of multiple training variables (e.g. load, volume, rest periods) to elicit desired adaptations [4]. Traditionally, resistance training is prescribed with a consistent external load (i.e. free weights or weight machine), which is moved across a full range of motion [5], and uses standardised rest periods between sets, with all sets of an exercise being completed before the execution of the subsequent exercise. While typical periodisation and prescription strategies (e.g. slight increases in training load every week) are effective for inducing physical adaptations, particularly in less experienced individuals [6], advanced methods of resistance training that manipulate key training variables (e.g. load, volume, rest periods) are commonly advocated as superior strategies for enhancing physical qualities and performance capacities [4, 6, 7]. However, there is limited evidence demonstrating the superiority of advanced methods over traditional resistance training within healthy adults [6–9]. Alternatively, resistance training prescription may need to be adjusted depending on contextual factors (e.g. group size, training schedule). Consequently, practitioners may implement advanced methods of resistance training that manipulate key training variables (e.g. load, volume, rest periods) in the aim of inducing superior adaptations or to suit the demands of training.

Advanced methods of resistance training can be introduced through the implementation of supplemental equipment that can alter the demands of an exercise. For example, accentuated eccentric loading employs either weight-releasers, bands, or dumbbells to overload the eccentric phase of an exercise [10, 11]. Through overloading the eccentric phase of a movement (which has relatively greater force production capabilities than the concentric phase), accentuated eccentric loading has been proposed to cause potentiation that improves the subsequent concentric contraction [11]. Alternatively, flywheel training can be performed with inertial devices that store the force produced in the concentric phase of the movement, which must be resisted, creating a more challenging eccentric phase than traditional resistance training [12]. Additionally, accommodating resistance can be implemented within training through the application of external bands or chains to alter force requirement within an exercise, increasing resistance during the portion of the exercise where force production capabilities are greatest [13]. However, these advanced methods of training may not be accessible or feasible for prescription within certain environments due to the equipment needed. Therefore, practitioners may use alternative advanced methods of restructuring training sessions to improve performance and adaptations without the need for supplemental equipment.

Practitioners can implement methods of training that restructure sets or session order. Sets can be restructured so that rest periods can be strategically implemented through cluster sets and rest redistribution (i.e. rest redistribution schemes), and this can allow the maintenance of kinetic and kinematic outputs. Cluster sets add short rest periods within a set of an exercise while the inter-set rest period is maintained [14]. Alternatively, rest redistribution is a method in which time is subtracted from the inter-set rest period and redistributed within the set [14]. Additionally, practitioners can rearrange the order of their session to take advantage of the potentiation caused by specific exercises. Potentiation complexes (i.e. complex or contrast training) improve the performance of a biomechanically similar subsequent exercises due to the potentiation caused by an initial exercise, also known as a conditioning activity [15]. Complex training structures alternate after each set between the conditioning activity and the subsequent exercise, whereas contrast training structures complete all sets of the conditioning activity prior to completing all sets of the subsequent exercise [15]. While these advanced methods of restructuring training can be used to enhance performance, there are also alternative methods of changing session structure that can aid in training efficiency.

It is important that the training prescribed by practitioners is not only effective in achieving desired performance outcomes, but also feasible and time efficient within the practical constraints of the training environment. Consequently, advanced methods of training have also been developed to maximise the training stimulus provided within a limited amount of time. For example, supersets involve the completion of two exercises consecutively (i.e. bench press and prone row) followed by a recovery, in a bid to improve session efficiency [16]. On the other hand, dropsets can be performed to increase the volume performed within a training session. When performing dropset training structures, exercises are usually taken within close proximity of (or to) momentary muscular failure (i.e. the point during a set where another repetition cannot be performed while maintaining adequate form), which is immediately followed by reductions in load before performing as many as reps as possible once again (with minimal/no rest between the change in weight) [17]. Therefore, supersets and dropsets can allow practitioners to provide an efficient and novel stimulus in comparison to traditional resistance training.

Throughout the literature, meta-analytical comparisons have been made between the adaptations seen from individual advanced methods and traditional resistance training [8, 9, 18]. Additionally, in certain cases, comparisons have been made between advanced methods (e.g. complex vs contrast training) [15]. However, no meta-analytical comparison has evaluated advanced resistance training methods collectively, rather than in isolation. Therefore, there is currently no understanding as to whether any of the advanced methods are superior to traditional resistance training, or to each other, in developing specific physical capacities or a broader range of adaptations. Consequently, this systematic review and Bayesian network meta-analysis aims to (1) compare advanced methods of resistance training prescription and their effects on strength, power, hypertrophy, and performance adaptations in healthy adults; (2) identify variables that may influence adaptations following specific resistance training methods; and (3) provide a rank order of advanced resistance training methods regarding their effectiveness for developing each physical capacity.

## Methods

### Search Strategy

This review was performed in line with the Preferred Reporting Items for Systematic Reviews and Meta-Analyses extension statement for network meta-analyses (PRISMA-NMA) [19]. A systematic review and Bayesian network meta-analysis protocol that includes the review question, search strategy, exclusion criteria, risk-of-bias assessment, and strategy for data synthesis was registered on 17th June 2024 with the Open Science Framework (https://osf.io/4b589). The academic databases SPORTDiscus, CINAHL, Scopus, PubMed, and MEDLINE were systematically searched in June 2024 to identify English-language peer-reviewed original research studies that investigated the effects of advanced resistance training methods on strength, power, hypertrophy, or performance measures. To ensure inclusion of the most recent evidence, the systematic search was updated in October 2025. Variance in database design led to slight differences in searches, with studies identified by searching abstracts, titles, and key words (found in ‘Online Supplemental Material 1’; see the electronic supplementary material). Additionally, the references of systematic reviews found within the search strategy (‘Online Supplemental Material 2’) were screened for eligibility. All search results were extracted and imported into Covidence reference manager (Covidence, Veritas Health Innovation, Melbourne, Australia).

### Selection Criteria

Duplicate studies were automatically removed by Covidence, and the titles and abstracts of the remaining studies were individually screened for relevance by two authors (NC and JW). Any disagreements in title and abstract screening were resolved by a third author (GM). Following title and abstract screening the remaining studies were then assessed for eligibility within their full text (NC and JW. To be eligible for inclusion, studies were required to (1) be original research investigations; (2) be full-text articles written in English; (3) be published in a peer-reviewed academic journal; (4) include healthy adults ≥ 18 and ≤ 50 years old; (5) compare at least two of eight unique resistance training conditions (see Sect. 2.3) in a randomised study design; (6) measure strength (i.e. dynamic 1–6 repetition maximum (RM), isometric test, isokinetic test), power (i.e. power output from vertical jumps, free weights, or plate loaded exercises), hypertrophy (i.e. muscle cross sectional-area, volume, or thickness from magnetic resonance imaging, ultrasonography, or a computed tomography scan), jump performance (i.e. jump height or distance), or sprint performance (i.e. time or velocity); (7) use standardised training methods between conditions (i.e. same exercises, load, repetitions); (8) have the majority (i.e. > 50%) of compound exercises implement the examined resistance training conditions; (9) have any external exercise (e.g. team sports training) performed by each condition; (10) have a training intervention lasting at least 6 weeks (i.e. allowing sufficient time for strength, power, hypertrophy, jump, or sprint adaptations); and (11) have two or more resistance training sessions per week. Studies were excluded for the following: (1) they combined two of the seven advanced resistance training methods; (2) they explicitly mentioned unsupervised resistance training (i.e. could not ensure correct execution of intervention and no additional resistance training that may influence outcome measures). Studies that clearly did not meet the inclusion criteria were removed. Any disagreements were resolved through discussion or with an additional author (GM).

### Data Extraction and Coding of Outcomes

Data from the included studies were then extracted by two authors (NC and JW). Arms of included studies were classified into traditional resistance training or one of seven advanced resistance training methods (refer to Table 1 for resistance training method definitions). Resistance training conditions were denoted with a three-character acronym (i.e. ACC = accommodating resistance; AEL = accentuated eccentric loading; DRO = dropsets; FLY = flywheel training; PTC = potentiation complexes; RRS = rest redistribution schemes; SUP = supersets; TRA = traditional resistance training). The data extracted from studies included participant and study characteristics (e.g. age, sex, sample size), resistance training condition details (e.g. training method, frequency, weekly volume load), and outcome measurements (a full list of the data extracted is available in ‘Online Supplemental Material 3’). In cases where the data were not reported numerically in a study, the study’s authors were contacted, or alternatively the data were extracted from graphs using WebPlotDigitizer (https://automeris.io/). Consistent with the guidelines of Cooper et al. [20] and previous sport science literature [21, 22], 30% of the included studies were randomly selected for re-examination to assess for potential coder drift. Agreement between authors was calculated by dividing the number of variables coded the same by the total number of variables. Acceptance required a mean agreement of 0.90 to avoid re-extraction entirely, and after this was met, only those with differing extracted data were checked and updated.

**Table 1:** Definitions of resistance training methods investigated

Condition	Abbreviation	Definition
Traditional resistance training	TRA	Traditional resistance training can be defined as training where the external load (i.e. free weights or weight-machine) is constant throughout the full range of motion (5), periods of rest are consistent between sets without additional rest within sets, and all sets of an exercise are completed before the execution of the subsequent exercise
Accommodating resistance training	ACC	ACC, also referred to throughout the literature as variable resistance, applies external resistance through bands or chains to alter the torque–time curves of an exercise (11). As a result, the resistance of an exercise increases during the portion of the range of motion where greater torque is able to be produced (11)
Potentiation complexes	PTC	Both complex and contrast training structures fall within the classification of PTC. PTC improves the performance of mechanically similar subsequent exercises due to the potentiation caused by an initial exercise, also known as a conditioning activity (9). Complex training structures alternate after each set between the conditioning activity and the subsequent exercise, whereas contrast training structures complete all sets of the conditioning activity prior to completing all sets of the subsequent exercise (13)
Accentuated eccentric loading	AEL	AEL uses either bands, dumbbells, or weight-releasers to overload the eccentric portion of an exercise (8, 9). It is known that force production capabilities are relatively greater in eccentric contractions when compared to concentric portions of the movement (11). Therefore, through overloading eccentric actions, it has been proposed that subsequent concentric performance is improved when using AEL, due to high threshold motor-unit recruitment (9)
Flywheel training	FLY	FLY devices store the energy produced during the concentric phase, which the athlete then has to resist throughout the eccentric portion of the movement, creating a more challenging eccentric contraction than traditional exercises (10)
Rest redistribution schemes	RRS	RRS can be implemented throughout individual exercises to maintain performance through either cluster sets or rest redistribution methods. Cluster sets add short rest periods within a set of an exercise, while rest redistribution is a method in which time is subtracted from the inter-set rest period and redistributed throughout the set (12)
Supersets	SUP	SUP involve the completion of two exercises consecutively (i.e. bench press and prone row) followed by a recovery, in a bid to improve session efficiency (14)
Dropsets	DRO	When performing DRO training structures exercises are typically taken to (or within close proximity of) momentary concentric failure, which is immediately followed by reductions in load before performing as many as reps as possible (with minimal/no rest between the change in weight) (15)

*Reps* repetitions

For outcome measurements, mean and standard deviation at pre- and post-intervention were extracted. When SD was unreported, SD was calculated using either standard errors, confidence intervals, *p* values, or *t* statistics [23]. When resistance training loads were reported as an RM, they were converted into a percentage of 1RM (%1RM) by using the equation %1RM = 100 − (RM(2.5)) [24]. When multiple measurements were reported for the same outcome measure, the highest-ranked measurement was extracted, as decided by a pre-determined hierarchy adapted from Currier et al. (see ‘Online Supplemental Material 4’) [23, 24]. Furthermore, if outcome measures were assessed at numerous timepoints (e.g. 4 weeks post-baseline and 8 weeks post-baseline), the timepoint furthest away from baseline was used [23, 24].

### Assessment of Reporting Quality

The within-study risk of bias was assessed independently by two reviewers (NC and JW) using the Cochrane Risk of Bias tool V.2 [25]. Criteria and questions were used to evaluate risk of bias for the ‘intention-to-treat’ effect. Articles were assessed at an outcome level (i.e. strength, power, jump, hypertrophy, and sprint) for the criteria pertaining to (1) randomisation processes; (2) deviations from the intended interventions; (3) missing outcome data; (4) measurement of the outcome; and (5) selection of the reported result. Each criterion was graded as low, some concerns, or high risk, which contributed to a risk-of-bias classification for each study. Any disagreements between the two authors were resolved through discussion.

### Quantitative Synthesis

As outcomes were measured using various tools and procedures (e.g. different outcomes and methods of assessing strength), standardised mean differences (SMDs) were calculated as the summary statistic [23]. The direction of effect was standardised between measures for each outcome variable so that positive values indicated greater adaptation for a training condition. Due to the nature of indirect comparisons throughout each network, nodes that included less than four papers were excluded, with the aim of maintaining statistical power [26]. Random-effects pairwise meta-analyses were performed to identify comparison-level heterogeneity, publication bias, outliers, and influential cases when multiple studies compared two conditions [24, 27, 28]. The *I*^*2*^ statistic was used to assess heterogeneity. Outliers were identified as studies with an SMD > 3.0 [29]. Publication bias was identified using visual inspection of funnel plots and Egger’s regression [30].

The network meta-analysis integrated all the direct evidence obtained from the systematic search, creating a network for each outcome (i.e. strength, power, hypertrophy, jump performance, sprint performance). The network meta-analysis developed for each outcome was fitted with a Bayesian framework using Markov chain Monte Carlo methods [28, 31], due to the ability to better account for complex networks in comparison to frequentist methods [32]. Four chains were run with vague priors [33]. A total of 50,000 iterations were included per chain, with the first 10,000 discarded as burn-in iterations, and a thinning interval of 10 for collected values [31]. Visual inspection of trace plots and density plots were used to evaluate convergence and the potential scale reduction factor [31, 34]. Fixed- and random-effects models were fit, with the more parsimonious model used for the analysis [24, 35]. Deviance information criterion (DIC) and posterior mean residual deviance were used to assess model fit [31, 35, 36]. Global inconsistencies were assessed by comparing DIC, model fit, and variance parameters of both the network meta-analysis and design-by-treatment interaction model [24, 37]. Additionally, the assumption of transitivity was visually assessed using age, sex, intervention duration, frequency, and relative weekly training volume load (i.e. average weekly value for volume × load × %1RM within outcome region of assessment) [38]. Relative effects were displayed using forest plots and league tables. The ranking probabilities of each condition were determined by assessing the surface under the cumulative ranking curve values. The results of the meta-analysis are presented as posterior SMDs and 95% credible intervals (CrIs).

Sensitivity analyses were performed to analyse the effect of outliers on relative effects, treatment rankings, and sources of network inconsistencies [23, 24]. The sensitivity analysis excluded any studies identified during pair-wise meta-analyses [23, 24]. To determine any additional factors that improved the fit of the developed model and altered the training condition effects, network meta-regressions were performed [23, 39]. Covariates for the network meta-regression included age, sex (percentage of participants that are male), resistance training experience, outcome measure region, intervention duration, training frequency, relative weekly training volume load, and whether or not training was taken to momentary muscular failure [24]. Multivariate imputations by chained equations (*n* imputations = 50) were used to manage missing data on covariates. Missing covariate values were only imputed if less than 10% of studies did not report the variable due to the unreliability of imputing greater percentages [24, 40]. If the percentage of missing covariate values was greater than 10%, the network meta-regression was not performed.

All statistical analysis for the study was performed with R Studio (version 2024.09.0) using the following packages: *escalc* (i.e. calculating SMD); *BUGSnet* (i.e. direct evidence pairwise meta-analyses to assess comparison-level heterogeneity, perform network meta-analyses, network meta-regressions, and consistency tests) [28]; *metafor* (i.e. test for publication bias); and *mice* (i.e. perform multiple imputations) [24, 41]. Data were visualised using *BUGSnet* [28] and *ggplot2* [42] packages, with additional figures being produced through NMAstudio (https://www.nmastudioapp.com/) software.

## Results

### Included Studies

**Fig. 1:**
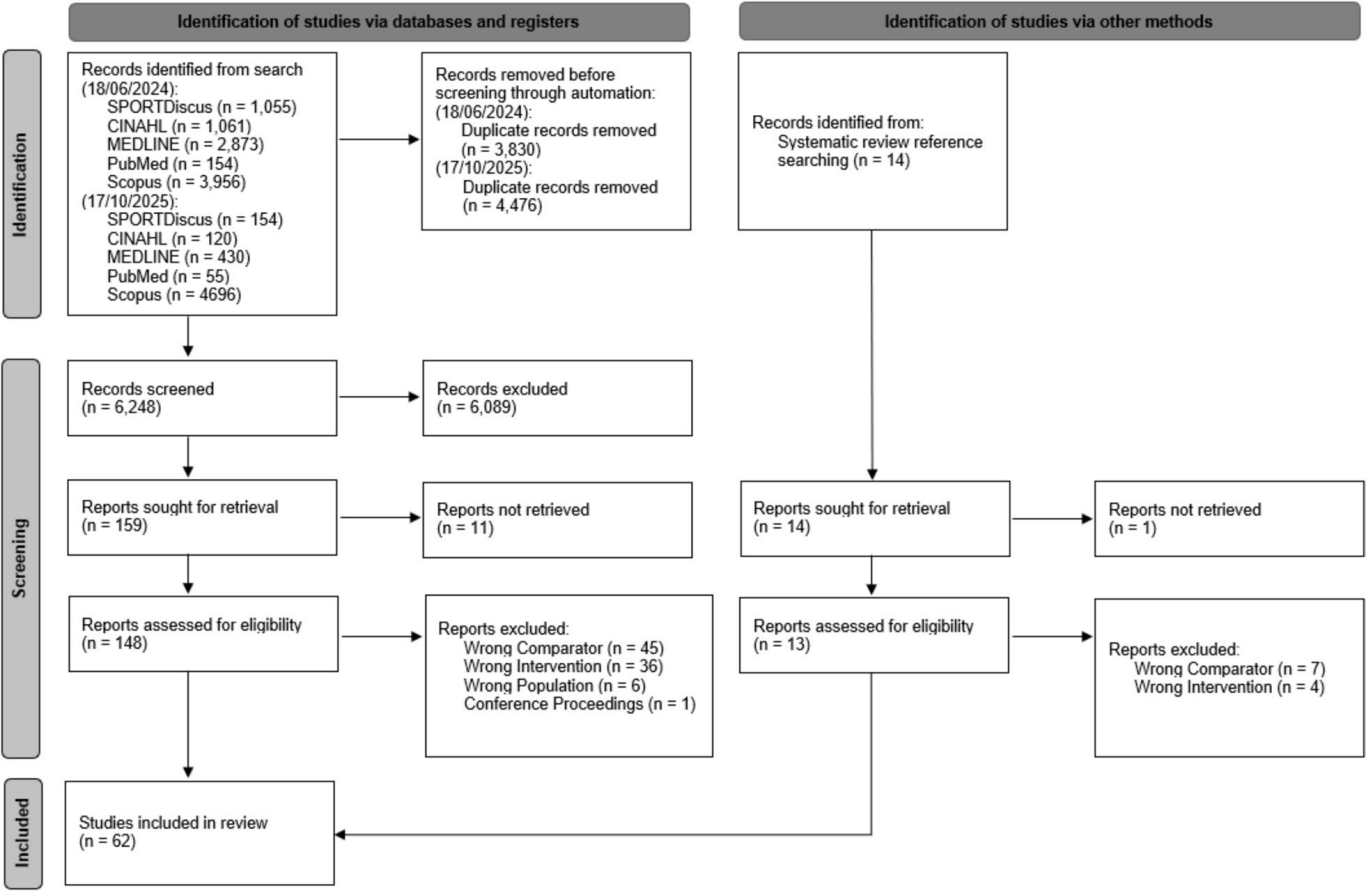
PRISMA-NMA flow diagram outlining inclusion and exclusion of studies across the screening process. *PRISMA-NMA* Preferred Reporting Items for Systematic Reviews and Meta-Analyses extension statement for network meta-analyses

**Fig. 2:**
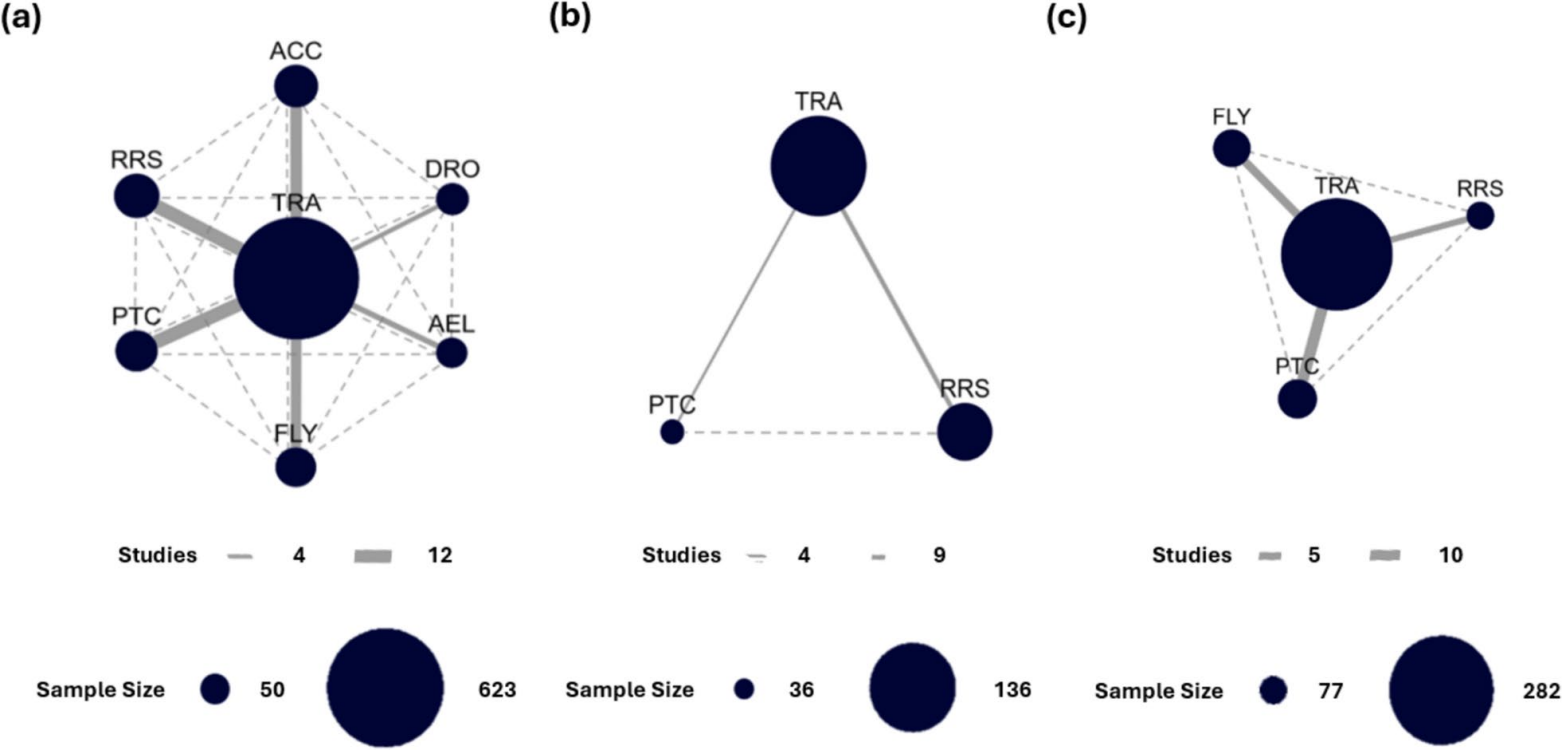
Network plots for strength (**a**), power (**b**), and jump performance (**c**) outcomes. Solid lines connecting training condition nodes represent direct comparisons from the literature. Dashed lines connecting condition nodes represent indirect comparisons calculated through the Bayesian network meta-analysis. The proportional size of nodes within each network demonstrates sample size, while the proportional thickness of the direct comparisons highlights the number of studies within each training condition. *ACC* accommodating resistance, *AEL* accentuated eccentric loading, *DRO* dropsets, *FLY* flywheel, *PTC* potentiation complexes, *RRS* rest redistribution schemes, *TRA* traditional resistance training,

Following the removal of duplicates, the systematic search resulted in a total of 6248 studies. Furthermore, the screening of systematic review reference lists resulted in an additional 14 studies sought out for screening. The titles and abstracts of the 6262 studies were reviewed, with 159 manuscripts sought out for full-text screening. The full-text screening process resulted in 62 studies that met the inclusion criteria (details of individual studies available in ‘Online Supplemental Material 5’). Specifically, 54 studies investigated strength outcomes, 18 studies investigated power outcomes, 31 studies investigated jump performance outcomes, 17 studies investigated hypertrophy outcomes, and 16 studies investigated sprint performance outcomes. Five studies included in the systematic review were not included in meta-analyses for particular outcomes, due to unavailable data [43–47]. Flow diagrams of the systematic search and network geometry charts for each physical outcome are demonstrated in Figs. 1 and 2, respectively.

### Research Reporting Quality

**Fig. 3:**
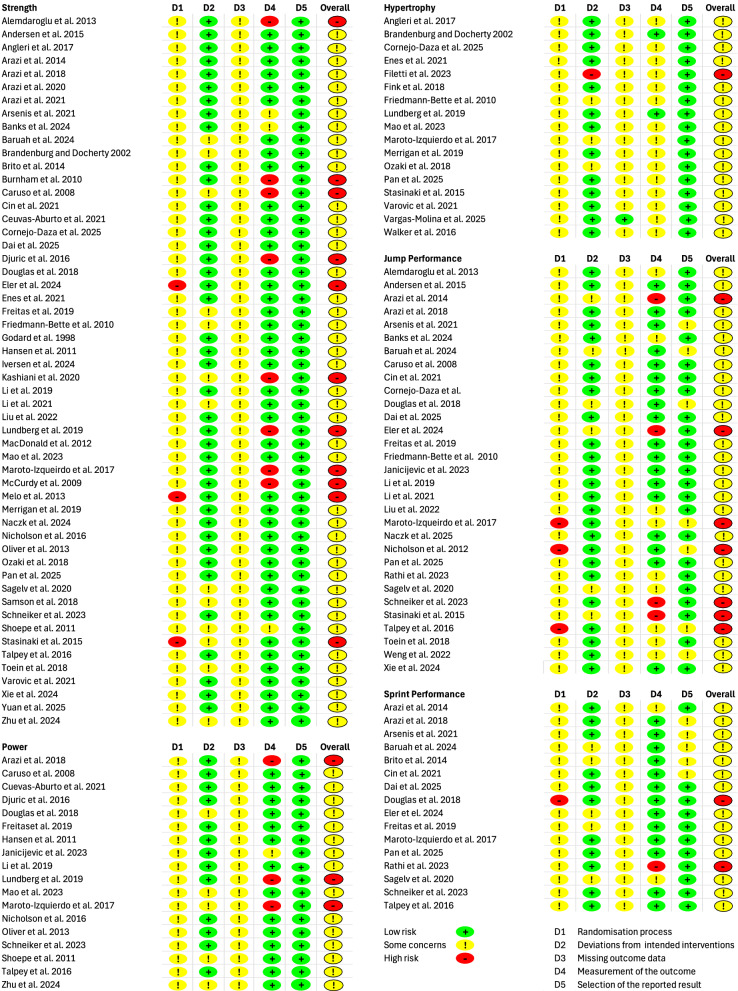
Risk-of-bias assessment for each outcome measure

Risk of bias ranged from ‘some concern’ to ‘high’ across manuscripts within strength (some concern = 43, high = 11), power (some concern = 15, high = 3), hypertrophy (some concern = 16, high = 1), jump performance (some concern = 24, high = 7), and sprint performance (some concern = 14, high = 2) outcomes. Assessments of risk of bias, including the selection for each criterion, can be found in Fig. 3.

### Comparison of Individual Advanced Methods to Traditional Resistance Training

**Fig. 4:**
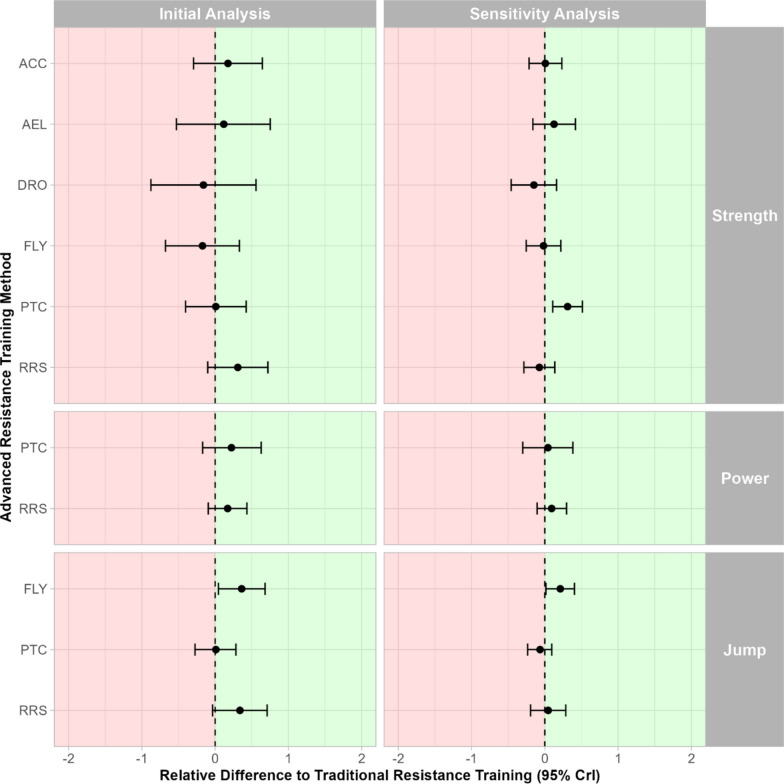
Forest plots demonstrating the estimates from each outcome network for the relative effect between each advanced method and traditional resistance training. Relative differences are expressed as SMD (95% CrI). The figure also demonstrates the effect of performing a sensitivity analysis and removing outliers for each network (right facet). The green shaded area represents an SMD in favour of the respective advanced resistance training method, while the red shaded area represents an SMD in favour of traditional resistance training. *ACC* accommodating resistance, *AEL* accentuated eccentric loading, *CrI* credible interval, *DRO* dropsets, *FLY* flywheel, *PTC* potentiation complexes, *RRS* rest redistribution schemes, *SMD* standardised mean difference

Each advanced method’s relative effect (i.e. methods with sufficient literature available) in comparison to traditional resistance training, both before and after performing a sensitivity analysis for each outcome network, is shown within Fig. 4. Supersets were not included within the analysis because only three eligible studies were retrieved from the systematic search. The SMD [95% CrI] for pairwise comparisons of strength outcomes ranged from − 0.17 [− 0.68 to 0.33] to 0.31 [− 0.10 to 0.72] across advanced methods, with rest redistribution schemes presenting the most favourable difference to traditional resistance training (SMD [95% CrI] = 0.31 [− 0.10 to 0.72]). However, no condition had a relative difference that had a 95% probability of being greater than traditional methods (i.e. 95% CrIs crossed zero). After performing a sensitivity analysis, the greatest difference was seen between potentiation complexes and traditional resistance training (SMD [95% CrI] = 0.31 [0.11–0.51]), presenting the only 95% CrI to not cross zero.

Two conditions were compared to traditional resistance training for power outcomes (i.e. potentiation complexes and rest redistribution schemes). It should be noted that the initial power analysis detected publication bias (*p* = 0.009); however, upon performing the sensitivity analysis, publication bias was accounted for (*p* = 0.188). Therefore, results for power outcomes were interpreted from the sensitivity analysis. Both comparisons to traditional training (i.e. potentiation complexes SMD [95% CrI] = 0.05 [− 0.34 to 0.45]; rest redistribution schemes SMD [95% CrI] = 0.09 [− 0.10 to 0.30]) had CrIs that crossed zero.

For jump performance, three conditions (i.e. flywheel training, potentiation complexes, and rest redistribution schemes) were compared to traditional resistance training, due to a lack of studies within other training methods. Publication bias was present upon initial analysis (*p* = 0.002), although, following the sensitivity analysis, publication bias was accounted for (*p* = 0.300). The SMD [95% CrI] ranged from − 0.07 [− 0.23 to 0.09] to 0.21 [0.01–0.40] for all pairwise comparisons between advanced methods and traditional resistance training for jump performance. The largest difference between conditions was seen between flywheel training and traditional resistance training, providing the only SMD with CrIs that did not cross zero (SMD [95% CrI] = 0.21 [0.01–0.40]).

All relative effects for the strength (initial analysis), power (sensitivity analysis), and jump outcomes (sensitivity analysis) can be found in Table 2, while the relative effects of the alternate analysis for each outcome can be found in ‘Online Supplemental Material 6’.

**Table 2:** Relative effects between all resistance training conditions within each network

Strength	RRS	ACC	AEL	PTC	TRA	DRO	FLY
RRS	**–**	− 0.13 (− 0.76 to 0.48)	− 0.19 (− 0.96 to 0.57)	− 0.30 (− 0.87 to 0.28)	− 0.31 (− 0.72 to 0.10)	− 0.47 (− 1.30 to 0.35)	− 0.48 (− 1.13 to 0.16)
ACC	0.13 (− 0.48 to 0.76)	**–**	− 0.06 (− 0.86 to 0.73)	− 0.17 (− 0.79 to 0.46)	− 0.18 (− 0.64 to 0.29)	− 0.33 (− 1.20 to 0.51)	− 0.35 (− 1.04 to 0.34)
AEL	0.19 (− 0.57 to 0.96)	0.06 (− 0.73 to 0.86)	**–**	− 0.11 (− 0.86 to 0.64)	− 0.12 (− 0.75 to 0.53)	− 0.28 (− 1.23 to 0.69)	− 0.29 (− 1.11 to 0.52)
PTC	0.30 (− 0.28 to 0.87)	0.17 (− 0.46 to 0.79)	0.11 (− 0.64 to 0.86)	**–**	− 0.01 (− 0.42 to 0.40)	− 0.17 (− 0.99 to 0.66)	− 0.18 (− 0.82 to 0.46)
TRA	0.31 (− 0.10 to 0.72)	0.18 (− 0.29 to 0.64)	0.12 (− 0.53 to 0.75)	0.01 (− 0.40 to 0.42)	**–**	− 0.16 (− 0.88 to 0.56)	− 0.17 (− 0.68 to 0.33)
DRO	0.47 (− 0.35 to 1.30)	0.33 (− 0.51 to 1.20)	0.28 (− 0.69 to 1.23)	0.17 (− 0.66 to 0.99)	0.16 (− 0.56 to 0.88)	**–**	− 0.01 (− 0.89 to 0.86)
FLY	0.48 (− 0.16 to 1.13)	0.35 (− 0.34 to 1.04)	0.29 (− 0.52 to 1.11)	0.18 (− 0.46 to 0.82)	0.17 (− 0.33 to 0.68)	0.01 (− 0.86 to 0.89)	**–**
Power	RRS	PTC	TRA				
RRS	**–**	− 0.05 (− 0.45 to 0.34)	− 0.09 (− 0.30 to 0.10)	**–**	**–**	**–**	**–**
PTC	0.05 (− 0.34 to 0.45)	**–**	− 0.04 (− 0.38 to 0.30)	**-**	**–**	**–**	**–**
TRA	0.09 (− 0.10 to 0.30)	0.04 (− 0.30 to 0.38)	**–**	**–**	**–**	**–**	**–**
Jump	FLY	RRS	TRA	PTC			
FLY	**–**	− 0.17 (− 0.48 to 0.15)	− **0.21 (− 0.40 to** − **0.01)**	− **0.28 (**− **0.53 to** − **0.03)**	**–**	**–**	**–**
RRS	0.17 (− 0.15 to 0.48)	**–**	− 0.05 (− 0.29 to 0.19)	− 0.11 (− 0.41 to 0.18)	**–**	**–**	**–**
TRA	**0.21 (0.01 to 0.40)**	0.05 (− 0.19 to 0.29)	**–**	− 0.07 (− 0.23 to 0.09)	**–**	**–**	**–**
PTC	**0.28 (0.03 to 0.53)**	0.11 (− 0.18 to 0.41)	0.07 (− 0.09 to 0.23)	**–**	**–**	**–**	**–**

Results for the strength network are from the initial analysis, while results for the power and jump networks are from the sensitivity analyses, due to publication bias. Relative effects are presented as SMD (95% CrI), with a positive effect favouring the condition in the column header. Results in bold highlight a 95% probability that the favoured condition presents a greater relative effect in developing the respective physical capacity

*ACC* accommodating resistance, *AEL* accentuated eccentric loading, *DRO* dropsets, *FLY* flywheel, *PTC* potentiation complexes, *RRS* rest redistribution schemes, *TRA* traditional resistance training

### Network Comparisons

Bayesian network meta-analyses were not able to be performed for hypertrophy and sprint performance, due to each network having less than four studies in at least three resistance training methods. However, relative effects between each resistance training method are displayed for strength, power, and jump performance in Table 1. For strength, the 95% CrI for all comparisons crossed zero. However, the sensitivity analysis resulted in five comparisons having 95% probability of yielding a greater effect: potentiation complexes versus accommodating resistance (SMD [95% CrI] = 0.30 [0.00–0.60]), potentiation complexes versus traditional resistance training (SMD [95% CrI] = 0.31 [0.11 –0.51]), potentiation complexes versus flywheel training (SMD [95% CrI] = 0.33 [0.02–0.64]), potentiation complexes versus rest redistribution schemes (SMD [95% CrI] = 0.38 [0.10–0.68]), and potentiation complexes versus dropsets (SMD [95% CrI] = 0.46 [0.09–0.83]). In the power networks, all 95% CrIs crossed zero. For jump performance, two comparisons resulted in a 95% probability of producing a greater effect: flywheel training versus traditional resistance training (SMD [95% CrI] = 0.21 [0.01–0.41]) and flywheel training versus potentiation complexes (SMD [95% CrI] = 0.28 [0.02–0.53]). All relative effects for alternate analyses in each outcome network can be found in ‘Online Supplemental Material 6’.

### Resistance Training Method Rankings

Ranking probabilities for every resistance training method within the strength, power, and jump networks, along with the effect of removing outliers, are shown within ‘Online Supplemental Material 7’. The strength network demonstrated that rest redistribution schemes (44.1%), accentuated eccentric loading (21.2%), and accommodating resistance (21.0%) were the three most likely methods to rank first in strength development, although, following a sensitivity analysis, the top three ranked conditions were potentiation complexes (82.4%), accentuated eccentric loading (14.3%), and accommodating resistance (1.6%). Alternatively, in the power network, rest redistribution schemes had the greatest probability of ranking first (56.1%). However, no meaningful differences (i.e. no SMD had 95% CrIs that did not cross zero) were seen within the strength and power networks. In the jump network, flywheel training had the greatest probability of ranking first (85.8%).

### Network Inconsistency

The DIC of the random effects model was not meaningfully different to the inconsistency model in the strength (i.e. DIC = 204.41 vs 204.68), power (i.e. DIC = 42.70 vs 43.06), and jump performance (i.e. DIC = 80.33 vs 80.27) networks. Full model fit comparisons can be found in ‘Online Supplemental Material 8’.

### Sensitivity Analyses

The results of the sensitivity analysis performed for each network can be found in ‘Online Supplemental Material 6’. The model fit was improved for the strength (i.e. DIC = 204.41 vs 172.92), power (i.e. DIC = 51.50 vs 42.70), and jump performance (i.e. DIC = 99.39 vs 80.33) networks following the sensitivity analysis (‘Online Supplemental Material 9’). Subsequently, the results of each network were adjusted, which has been discussed in previous sections. Due to the publication bias that was present, the interpretation of results for power and jump performance was based on the sensitivity analysis.

### Network Meta-Regressions

The results from each network meta-regression are shown in ‘Online Supplemental Material 10’**.** The meta-regression for sex (i.e. percentage of male participants) within the strength network demonstrated rest redistribution schemes to have a meaningful difference to traditional resistance training (SMD [95% CrI] = 0.38 [0.02–0.74]) as a smaller percentage of males were included within studies. Whereas in the jump performance network, meta-regressions revealed increased training frequency to favour rest redistribution schemes (SMD [95% CrI] = 0.21 [0.00–0.42]) and traditional resistance training (SMD [95% CrI] = 0.28 [0.01–0.55]) in comparison to flywheel training, while greater intervention durations and relative weekly volume loads removed any meaningful differences between resistance training methods. No covariates resulted in meaningful differences between resistance training methods throughout the power network. Furthermore, there was no substantial difference in model fit when comparing the initial analysis to the implementation of all covariates for strength, power, and jump performance adaptations (‘Online Supplemental Material 11’).

## Discussion

**Fig. 5:**
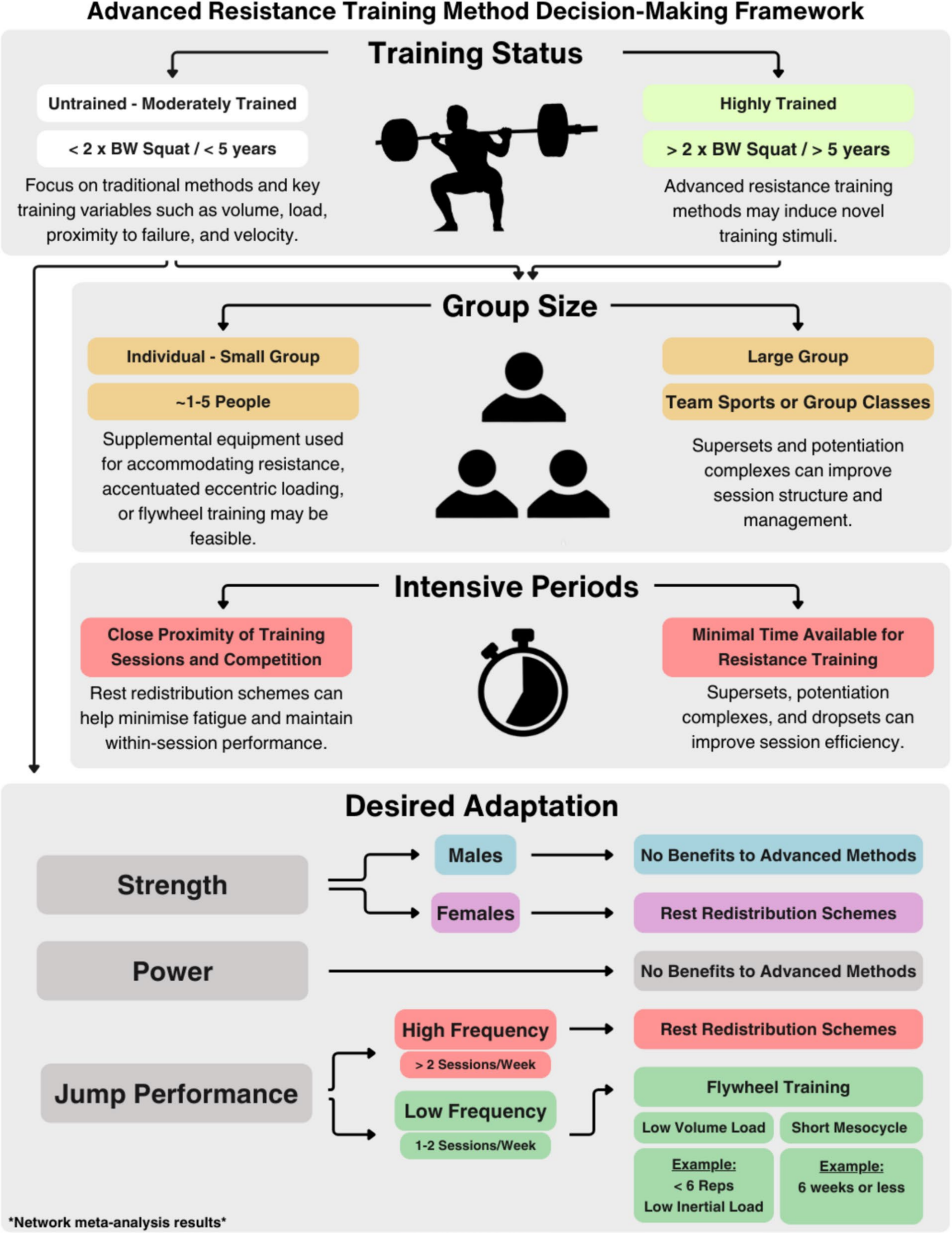
Decision-making framework for the implementation of advanced resistance training methods. *BW* bodyweight, *reps* repetitions

The aims of this study were to (1) compare advanced methods of resistance training prescription and their effects on strength, power, hypertrophy, and performance adaptations in healthy adults; (2) identify variables that may influence adaptations following specific resistance training methods; and (3) provide a rank order of advanced resistance training methods in their effectiveness for developing each physical capacity. Sixty-two papers were included that investigated strength, power, hypertrophy, jump, or sprint adaptations for traditional and advanced methods of resistance training. The Bayesian network meta-analyses demonstrated that advanced methods have no clear added benefit in comparison to traditional resistance training for developing strength and power, although network meta-regressions highlighted that rest redistribution schemes may be more effective for developing strength in females in comparison to males. While rank orders were also calculated for each resistance training method’s effectiveness in improving strength and power, these rankings should be interpreted with caution due to the lack of meaningful differences seen when comparing the training methods within each network. The network meta-analysis for jump performance revealed flywheel training to be the most effective resistance training method—although meta-regressions revealed flywheel training may be most beneficial for improving jump performance with lower training volume loads and shorter intervention durations, with rest redistribution schemes becoming more effective with higher training frequency. However, considering the lack of clear benefit for advanced training methods over traditional forms of resistance training for the development of physical capacities within healthy adults, practitioners are strongly recommended to prioritise the implementation of traditional training methods and emphasise the manipulation of fundamental training variables (e.g. load, volume) that have been shown to have clear, demonstrable, and proven effects on physical adaptations and performance [48, 49]. Figure 5 provides a decision-making framework that can help inform appropriate prescription of advanced resistance training methods.

### Strength

Of the 54 studies that investigated strength outcomes [5, 44, 45, 50–98], 42 studies showed no significant difference between advanced and traditional methods [5, 44, 45, 50–52, 54, 55, 58–66, 68, 70–80, 82, 83, 85, 86, 90–92, 94–97], eight studies showed significantly greater adaptations following advanced methods of training (accommodating resistance = 2; accentuated eccentric loading = 1; flywheel training = 1, rest redistribution schemes = 4) [53, 56, 57, 81, 87–89, 98], and four studies demonstrated greater adaptations following traditional resistance training (flywheel training = 3; rest redistribution schemes = 1) [67, 69, 84, 93]. The most researched comparisons between methods were potentiation complexes versus traditional resistance training (*n* = 12) and rest redistribution schemes versus traditional resistance training (*n* = 12). The training method with the fewest number of papers that investigated strength was supersets (*n* = 3). While supersets were not included in the meta-analytical comparisons, all studies highlighted no significant differences in strength adaptations when compared to traditional methods, suggesting supersets to be a time-effective method of developing strength [92, 99, 100].

The meta-analytical comparisons of each advanced method to traditional resistance training demonstrated no clear differences in strength adaptations. Following the removal of outliers through a sensitivity analysis, the results demonstrated lower variability and suggested that potentiation complexes provided greater strength adaptations when compared to traditional methods. However, the differences seen in strength adaptations may be due to subtle differences in study design and how the potentiation complex and traditional conditions were implemented. In multiple studies, potentiation complexes performed greater within-session volume load than traditional training (i.e. potentiation complexes = exercises performed in traditional condition + potentiation exercises) [45, 75, 90], which may have caused superior strength improvements [101, 102]. Alternatively, Schneiker et al. [76] prescribed a mesocycle of strength exercises (i.e. squats and deadlifts using 85% 1RM) followed by a mesocycle of power exercises (i.e. squat jumps and clean pulls using 30% 1RM) within the traditional condition. Therefore, the traditional resistance training condition was not exposed to strength exercises (i.e. compound exercise with heavier loading schemes) in the second half of the intervention, which may have led to diminished levels of strength, while the potentiation complex condition performed both the strength and power exercises across the entire study. [76]. Subsequently, the improvements seen from performing potentiation complexes may have been due to the continual exposure to strength exercises and the specificity that can be seen in adaptations from the training performed [103, 104].

The network meta-analysis found none of the advanced methods to be superior to any other for the development of strength. It should be noted that indirect comparisons between advanced methods are calculated from their respective direct comparisons to traditional resistance training. Consequently, the lack of differences seen between advanced methods throughout the network meta-analysis are due to the trivial relative effects when comparing each method to traditional training. However, the sensitivity analysis results suggested potentiation complexes to be more beneficial for improving strength in comparison to dropsets, rest redistribution schemes, flywheel training, and accommodating resistance. In the dropset interventions, participants often only performed one to two isolation exercises (e.g. leg extension or dumbbell curls) [63–65], while potentiation complex interventions typically implemented a greater number of exercises that were multi-joint (e.g. free-weight barbell back squat) and allowed for heavier external loads. Therefore, the estimated differences between potentiation complexes and dropsets may be due to greater training volume load and overall stimulus [101, 102, 105]. Similarly, greater strength adaptations may have been observed in potentiation complexes compared to the rest redistribution scheme interventions due to potentiation complexes often using heavier loads within their conditioning activities. Additionally, while rest redistribution scheme interventions often implemented the same exercises and training volume between conditions, this was not always the case for potentiation complex interventions. Alternatively, the movement demands of the conditioning activities in potentiation complex training programmes typically shared greater similarity with testing exercises in comparison to the differing movement demands of flywheel training (i.e. difficulty of exercise dictated by the speed of the concentric phase) and accommodating resistance (i.e. altered force production requirements). Thus, exposure to greater loads and exercise specificity may help to explain the current results.

Network meta-regressions for strength revealed an effect of sex on adaptation. In fact, rest redistribution schemes became more effective than traditional resistance training as a greater percentage of participants were female. Females have been shown to exhibit greater fatigue resistance [106], which can be associated with their higher proportions and cross-sectional areas of type I muscle fibres in comparison to males [107], although it should be noted that this difference in fatiguability is mitigated for high velocity and near-maximal contractions [106]. Nonetheless, rest redistribution schemes may be particularly beneficial for females, due to their physiological capacity to sustain kinematic outputs, which has been shown to support the development of strength [108]. Therefore, the effect of sex observed in the meta-regression may be explained by females’ enhanced fatigue resistance used in conjunction with training strategies that minimise within-session fatigue and maintain performance, such as rest redistribution schemes (108).

### Power

Across the 18 studies that investigated power-related outcomes [52, 56, 58, 67, 68, 71, 73, 76, 78–80, 82, 84–87, 95, 109], 14 showed no significant differences between advanced methods and traditional resistance training [52, 58, 67, 68, 71, 73, 76, 78, 82, 84–86, 95, 109], while four demonstrated significantly greater adaptations following advanced methods (accommodating resistance = 1; rest redistribution = 3) [56, 79, 80, 87]. There were no studies that investigated power outcomes following either dropsets or supersets. Additionally, while accommodating resistance (*n* = 2), accentuated eccentric loading (*n* = 1), and flywheel training (*n* = 3) had investigations that met the inclusion criteria, not enough studies were retrieved to include within the statistical analysis. The two studies that investigated accommodating resistance found conflicting results, with Djuric et al. [52] showing no difference to traditional resistance training and Shoepe et al. [56] highlighting greater improvements following an accommodating resistance intervention. However, more pronounced differences between training methods may have been due to the longer intervention implemented by Shoepe et al. [56] (i.e. 24- vs 8-week training period) [80]. Alternatively, Douglas et al. [58] was the only study to investigate power adaptations following accentuated eccentric loading and traditional training, reporting unclear differences within the first 4-week mesocycle (i.e. performing ‘slow-tempo’ eccentrics) and greater improvements from traditional resistance training in the second 4-week mesocycle (i.e. performing ‘fast-tempo’ eccentrics). It was proposed that greater fatigue caused within the accentuated eccentric loading condition may have affected subsequent power adaptations [58]. Meanwhile, both studies investigating flywheel versus traditional resistance training demonstrated similar power adaptations between the methods [67, 68].

There were no meaningful differences seen for the development of power when comparing potentiation complexes and rest redistribution schemes to traditional resistance training. When investigating power adaptations following potentiation complexes and traditional resistance training, all four studies found no significant differences [71, 73, 76, 78], which may be due to the methodological design of individual studies. For example, the only difference between potentiation complexes and traditional training in the intervention performed by Talpey et al. [78] was exercise order (i.e. traditional = ballistic exercise followed by heavy compound exercise; potentiation complexes = heavy compound exercise followed by ballistic exercise). Alternatively, within the study by Freitas et al. [71], the participants in the traditional condition performed exercises using a load proposed to maximise mechanical power, while the second (power-focused) mesocycle of Schneiker et al.’s [76] traditional intervention involved performing squat jumps and clean pulls using 30% 1RM of their squat and deadlift, respectively. Therefore, through using loads within traditional resistance training that optimise power output, potential benefits from potentiation complexes may have been masked. Similarly, there was no meaningful difference found between rest redistribution schemes and traditional resistance training.

Due to half of the studies not reporting resistance training experience when investigating power outcomes, a network meta-regression was unable to be performed. However, trends associated with resistance training experience and power adaptations following rest redistribution schemes have been highlighted throughout the literature. Of the nine studies that assessed rest redistribution schemes versus traditional resistance training, three studies demonstrated greater improvements in power output following rest redistribution schemes [79, 80, 87]. The enhanced adaptations seen within these studies may be due to the resistance training experience of the participants involved, as relatively untrained individuals are likely to experience similar adaptations irrespective of the method of training used [6]. Throughout the studies that investigated rest redistribution schemes versus traditional methods, the participants of those that highlighted favourable results following rest redistribution schemes had the greatest resistance training experience (i.e. Arazi et al. [79] = 5.5 years; Arazi et al. [80] = 3 years; Oliver et al. [87] = 6 years). Therefore, similarities between rest redistribution schemes and traditional resistance training in other studies may be due to the inclusion of relatively untrained participants. However, these differences should be interpreted cautiously due to the inability to perform a meta-regression for the resistance training experience of participants.

### Jump Performance

Throughout the 31 studies that investigated jump performance outcomes [5, 43, 44, 47, 58, 60, 66, 67, 69–74, 76–79, 81, 83, 86, 109–111], there were 22 studies that highlighted no significant difference between advanced methods and traditional resistance training [5, 44, 60, 66, 67, 69–74, 78, 86, 109, 111] and nine studies that highlighted significantly greater adaptations following advanced methods of resistance training (accentuated eccentric loading = 2; flywheel training = 2; potentiation complexes = 2; rest redistribution schemes = 3) [43, 47, 58, 76, 77, 79, 81, 83, 110]. No studies assessing jump performance following dropsets were included. Three studies examined the effects of accommodating resistance versus traditional resistance training and showed similar improvements in jump performance from both methods [5, 91, 96]. Alternatively, three studies evaluated differences in jump adaptations between accentuated eccentric loading and traditional resistance training [58, 60]. Both Douglas et al. [58] and Friedmann-Bette et al. [43] showed greater improvements in jump performance following accentuated eccentric loading. Furthermore, Douglas et al. [58] highlighted variations in adaptations depending on the mesocycle of the training intervention. Specifically, changes in drop jump reactive strength index and ground contact time were superior after performing a mesocycle of fast-tempo accentuated eccentric loading in comparison to traditional resistance training. Eccentric (i.e. braking) force production has been shown to be an important determinant of ground contact time in the drop jump exercise [58]. Therefore, it was suggested that the greater adaptations were due to the accentuated eccentric loading condition performing drop jumps with a faster, accentuated eccentric contraction throughout the mesocycle [58]. Meanwhile, García-Orea et al. [99] outlined similar changes in countermovement jump performance following supersets and traditional training, once again outlining the efficiency seen from implementing supersets within resistance training programmes.

When comparing resistance training methods within the network meta-analysis, flywheel training was shown to be more effective in developing jump performance than traditional resistance training and potentiation complexes. Both Maroto-Izquierdo et al. [47] and Weng et al. [110] demonstrated significantly greater adaptations from flywheel interventions in comparison to traditional resistance training. It was suggested that the demand of the eccentric contraction may have improved the muscle’s ability to utilise elastic energy during the stretch–shortening cycle [47, 110]. Moreover, flywheel training may utilise the stretch–shortening cycle with greater load more consistently than the bodyweight plyometrics typically seen in potentiation complexes. Collectively, these findings suggest that the increased eccentric demands within flywheel training may provide a more potent and consistent stimulus for enhancing stretch–shortening cycle function, and subsequently jump performance.

The meta-regressions completed for the jump performance network revealed numerous covariates affected the comparisons between resistance training methods. While the network meta-analysis ranked flywheel training the highest for improving jump performance, network meta-regressions showed that longer interventions and greater training volume loads removed meaningful differences between resistance training methods. The diminishing differences between resistance training methods with extended periods of training may be related to the training experience of participants. Most studies within the network meta-analysis included untrained to moderately trained individuals. For novice lifters, early gains in strength are primarily driven by neural adaptations [112]. Eccentric loading, which is exemplified by flywheel training, is known for producing greater neural adaptations in comparison to concentric and isometric contractions [113]. Therefore, the greater neural adaptations produced by the eccentric overload of flywheel training may have improved strength adaptations in shorter interventions. Alternatively, greater training volume load was also shown to reduce any benefits of flywheel training for jump performance, which is likely due to the reduced kinematics of heavier inertial loads and the greater fatigue produced from increased volume load. Greater total training volume is a main determinant in fatigue responses [114], and likely increased within-session fatigue and reduced kinematics that underpin jump performance (i.e. velocity and power) when performing flywheel training [115].

Network meta-regressions for jump performance also revealed that as training frequency increased, rest redistribution schemes and traditional resistance training were more beneficial for developing jump performance than flywheel training. Even though the network meta-analysis originally revealed flywheel training to be the most effective resistance training method for improving jump performance, the eccentric demand of the training method may lead to increased muscle damage [116] and, subsequently, worse recovery. Consequently, if flywheel training is prescribed with high frequency, sessions may be performed under fatigue, resulting in reduced within-session kinematics. Alternatively, rest redistribution schemes have been shown to reduce fatigue and maintain kinematic outputs [117]. Therefore, more effective development of jump performance in periods of higher training frequency with rest redistribution schemes and traditional resistance training is likely due to the ability to maintain performance and mitigate fatigue in comparison to the fatigue produced by the eccentric demand of flywheel training.

### Hypertrophy

Of the 17 studies that investigated hypertrophy-related outcomes [43, 47, 57, 61–65, 68, 77, 85, 91, 98, 100, 118–120], 13 studies demonstrated no significant differences between advanced methods and traditional training [43, 57, 61–63, 65, 68, 85, 91, 98, 100, 119, 120], while four studies (dropsets = 2; flywheel training = 1; potentiation complexes = 1) presented superior hypertrophic adaptations from an advanced method of resistance training [47, 64, 77, 118]. The most common comparisons between methods were traditional resistance training versus dropsets (*n* = 5) and traditional resistance training versus flywheel training (*n* = 3). Although there were no studies that investigated hypertrophy adaptations following accommodating resistance and supersets, it should be noted that no form of training consistently produced greater improvements in muscle hypertrophy. Meta-analytical comparisons could not be made between various advanced methods of resistance training for hypertrophy adaptations, due to the lack of literature available. However, differences between traditional resistance training and individual advanced methods can be evaluated.

Advanced methods of resistance training, such as dropsets, are often implemented to target hypertrophic adaptations by aiming to increase training volume and efficiency [121]. Enes et al. [62] compared dropsets to traditional resistance training and demonstrated that improvements in muscular hypertrophy were not significantly different. It was proposed that similarities in adaptations were due to the equated training volume across conditions, creating similar mechanical work [62]. Additionally, Ozaki et al. [63] demonstrated that training to momentary muscular failure in both traditional training and dropsets resulted in comparable hypertrophic adaptations, which may have been attributable to similar motor unit recruitment as a result of performing both conditions to failure [63, 122, 123]. It should be noted, though, that Fink et al. [118] also performed training to momentary muscular failure in both conditions and found superior hypertrophy from completing dropsets [118]. While our study did not perform a pairwise comparison between dropsets and traditional resistance training, the findings of our systematic review align with those of a previous meta-analysis comparing the two methods [124], highlighting similar hypertrophy adaptations.

Numerous studies have examined muscular hypertrophy following flywheel and traditional resistance training interventions [47, 68, 119]. Both Filetti et al. [119] and Maroto-Izquierdo et al. [47] investigated regional measures of lower limb hypertrophy and demonstrated superior adaptations following flywheel training. In particular, Filetti et al. [119] showed significantly greater vastus lateralis thickness in proximal, medial, and distal measurements [119]. Additionally, Maroto-Izquierdo et al. [47] showed greater vastus lateralis and rectus femoris hypertrophy from flywheel training, and superior vastus medialis hypertrophy from traditional resistance training [47]. Alternatively, Lundberg et al. [68] found similar adaptations in quadriceps femoris hypertrophy (i.e. all four muscles) between flywheel and traditional training [68]. The minimal amount of literature and conflicting findings that are currently available for not just flywheel training but all advanced resistance training methods highlight the need for further research into hypertrophy adaptations.

### Sprint Performance

Sixteen studies compared the sprint adaptations of an advanced method and traditional resistance training [45, 47, 58, 66, 69–71, 76, 78, 79, 81, 83, 90, 91, 96, 111], with 13 showing no significant difference between conditions [45, 66, 69–71, 76, 78, 79, 83, 90, 91, 96, 111]. There were no investigations that assessed sprint performance following dropsets or supersets, whereas the most frequent comparison investigated was potentiation complexes versus traditional resistance training (*n* = 7). A previous meta-analysis performed by Thapa et al. [125] demonstrated potentiation complexes to be more effective than traditional resistance training for developing 20-m sprint performance, but not 10- or 30- to 60-m sprint performance. However, none of the studies investigated within our systematic review demonstrated that potentiation complexes resulted in superior sprint performance improvements in comparison to traditional resistance training. The differences presented between the findings of this systematic review and previous meta-analyses are likely due to the stricter inclusion criteria and, subsequently, the smaller number of studies included in this paper.

Only three interventions highlighted a significant difference between potentiation complexes and traditional resistance training [47, 58, 81]. Cin et al. [81] and Maroto-Izquierdo et al. [47] demonstrated superior improvements in 20-m sprint times when comparing rest redistribution schemes and flywheel training to traditional resistance training, respectively [47, 81]. Alternatively, Douglas et al. [58] presented greater improvements in 40-m sprint performance after undertaking an accentuated eccentric loading intervention in comparison to traditional resistance training. It was proposed that the superior sprint adaptations may be due to increased tendon stiffness and upregulated muscle collagen synthesis rates caused by accentuated eccentric loading [58]. However, no meaningful differences between training methods were consistently found across the available literature.

Multiple studies investigating sprint adaptations suggested that similarities between advanced methods (rest redistribution schemes = 2; potentiation complexes = 3) and traditional resistance training were due to the significant relationship between strength and sprint performance [45, 76, 79, 83, 90]. Consequently, the comparable improvements across multiple studies in sprint performance could have been due to the minimal differences seen in strength adaptations following advanced methods and traditional training. Alternatively, Rathi et al. [111] suggested the incorporation of too many potentiation complexes within sessions, and the subsequent residual fatigue produced, affected performance and adaptation, minimising any potential benefits of potentiation complexes in comparison to traditional resistance training—whereas, across the three studies investigating flywheel versus traditional training, no consistent meaningful difference was presented.

### Limitations

Although this is the first systematic review and Bayesian network meta-analysis to compare the effects of different advanced resistance training methods on strength, power, hypertrophy, and performance adaptations in healthy adults, it is not without its limitations. First, the advanced methods of resistance training investigated throughout this study may be more effective with highly trained individuals, to provide a novel stimulus and further induce adaptation [6, 7]. However, the average resistance training age (i.e. from studies that reported a numerical value for resistance training age) for participants was 1.9 years, which is a relatively untrained level. Second, reporting of resistance training experience throughout the literature remains inconsistent, and definitions are often vague, using terms such as ‘recreationally trained’ or ‘physically active’ [44, 52]. In addition, those with minimal experience can often encounter similar improvements in physical capacities regardless of the resistance training method [6], potentially explaining the similarities in adaptations between conditions when studies investigated untrained or novice lifters. Unfortunately, a network meta-regression was unable to be performed, due to the high percentage of studies not reporting resistance training experience. Third, due to the nature of certain resistance training methods (e.g. flywheel training, potentiation complexes) and reporting of loads (i.e. RM prescription), relative weekly volume load often required values to be estimated. As it is known training volume can influence adaptation [101, 102], its effect on adaptations between conditions should be interpreted cautiously, due to approximations made when performing calculations. Fourth, pairwise comparisons were not performed for hypertrophy (i.e. dropsets vs traditional resistance training) and sprint outcomes (i.e. potentiation complexes vs traditional resistance training), due to the *BUGSnet* package needing the inclusion of three conditions, although systematic reviews were still performed and compared to respective meta-analyses. Fifth, while inclusion criteria for studies aimed to mitigate differences in studies, training variables that are known to influence adaptation (e.g. volume, load, intent, proximity to momentary muscular failure, training experience) varied across resistance training methods and studies [101, 102, 105, 122, 123, 126], potentially affecting the assumption of transitivity [127]. Finally, risk-of-bias assessments indicated that most included studies were rated as having ‘some concern’, with several classified as ‘high’ risk, which should be taken into consideration when interpreting the results of the network meta-analyses performed.

### Practical Considerations and Future Recommendations

The findings from this review highlight that advanced methods of resistance training may not be superior to traditional methods for developing overall physical performance. However, flywheel training was shown to be superior for inducing jump adaptations. Furthermore, it was demonstrated that intervention duration, training volume load, and frequency can influence the effectiveness of implementing flywheel training, as well as rest redistribution schemes, for improving jump performance. Therefore, during periods of lower training frequency (e.g. ≤ two sessions per week), practitioners aiming to improve jump performance should prescribe flywheel training across shorter mesocycles (e.g. ≤ 6 weeks), with fewer repetitions (e.g. ≤ six repetitions) and lighter inertial loads, whereas, in mesocycles with more frequent training (e.g. > two sessions per week), practitioners may prescribe rest redistribution schemes to minimise fatigue, maintain kinematics, and improve jump performance adaptations.

While long-term improvements in strength, power, hypertrophy, and performance outcomes are crucial for the development of clients and athletes, factors such as training schedules, load management, and athlete wellbeing are also important for practitioners to consider. Throughout busy training schedules, the management of training load and subsequent fatigue within micro- and mesocycles is pivotal in allowing athletes to perform (e.g. season fixtures) and avoid overtraining or injury [128, 129]. Consequently, practitioners may wish to implement methods that minimise the fatigue caused by resistance training when internal and external load from other activities (e.g. sport-specific training or fixtures) are high. For example, rest redistribution schemes have consistently been demonstrated to reduce mechanical, metabolic, and perceptual fatigue in comparison to traditional resistance training [130], whereas, an advanced method such as dropsets is designed to efficiently fatigue the involved musculature [131]. However, when comparing perceptual and metabolic, but not neuromuscular, fatigue, flywheel training and accentuated eccentric loading were shown to induce greater demands than dropsets and traditional training [132]. Alternatively, implementing supersets can reduce session duration, which may be an effective tool during busy schedules, although athletes may require longer periods for recovery [16]. Therefore, practitioners must assess the goals and needs of clients and athletes and their respective training cycles to select the appropriate resistance training method to implement.

Even though individual advanced resistance training methods could prove beneficial for acute and short-term training goals, the combination of multiple methods may be more effective. Some advanced methods implement supplemental equipment and adjust loads, whereas others manipulate session structure, providing scope to use methods in conjunction to potentially elicit greater responses. For instance, Masel and Maciejczyk [133] highlighted that the incorporation of accommodating resistance into a potentiation complex of trap-bar deadlifts and squat jumps (i.e. 15% elastic resistance when performing trap-bar deadlifts) significantly improved acute performance. Alternatively, it has been suggested that accentuated eccentric loading (when using weight-releasers in an exercise such as back squats) could be used in conjunction with rest redistribution schemes within a functional overreaching block to increase volume load and kinematic outputs [134]. In addition, the implementation of flywheel training as a conditioning activity has been shown to produce greater acute post-activation performance enhancement [135], and superior strength, power, jump, and hypertrophy adaptations in comparison to using traditional exercises throughout potentiation complex interventions [136]. The literature demonstrates that advanced methods (or a combination of them) may be more effective in achieving acute and short-term training goals, which may also impact long-term improvements. However, further research is needed to fully elucidate any potential benefits.

When performing future research, it should be understood that advanced methods of resistance training are likely to be most effective at inducing superior adaptations within highly trained athletes. However, the majority of research to date has involved individuals who are relatively untrained or has not reported the resistance training experience and relative strength of participants. Additionally, the current literature is limited in quantity, especially for outcomes such as power output, hypertrophy, and sprint performance. Therefore, it is necessary for future research into advanced methods of resistance training to not only include highly trained individuals (e.g. > 5 years resistance training experience or a relative measure of strength such as ≥ 2 × bodyweight back squat), but to also port their resistance training experience or relative strength, and assess these participants across a comprehensive range of outcome measures conducted using robust methods.

## Conclusion

This systematic review and Bayesian network meta-analysis highlights the minimal differences in the adaptations seen following traditional and advanced methods of resistance training. While it was shown that flywheel training is superior for developing jump performance, and rest redistribution schemes may be more effective for developing strength in females in comparison to males, there is a lack of compelling evidence to support the notion that advanced methods provide superior adaptations for healthy adults—although, advanced resistance training methods may better suit certain goals of training. Therefore, practitioners should be mindful of how they prescribe resistance training, and if they choose to implement an advanced method, they should consider the needs of the individual, the training cycle, and other training variables that may affect short-term responses as well as chronic adaptations. However, the limitations of the literature should be noted, and future studies should recruit participants with higher relative strength and training ages, while also assessing a comprehensive range of outcome metrics derived from rigorous methodology.

## Supplementary Information

Below is the link to the electronic supplementary material.

Supplementary file1 (DOCX 43 KB)

Supplementary file2 (DOCX 73 KB)

Supplementary file3 (DOCX 20 KB)

Supplementary file4 (DOCX 33 KB)

Supplementary file5 (DOCX 22 KB)

Supplementary file6 (DOCX 28 KB)

Supplementary file7 (DOCX 119 KB)

Supplementary file8 (DOCX 27 KB)

Supplementary file9 (DOCX 934 KB)

Supplementary file10 (DOCX 59 KB)

Supplementary file11 (DOCX 61 KB)

## Declarations

### Availability of data and material

All data and material reported in this systematic review and Bayesian network meta-analysis are from peer-reviewed publications.
