# Supplementary material for: The Effects of Advanced Resistance Training Prescription Methods on Strength, Power, Hypertrophy, and Performance Adaptations in Healthy Adults: A Systematic Review and Bayesian Network Meta-analysis
Source: Sports Med. 2026 Apr 8;56(8):1955–77. doi: 10.1007/s40279-026-02428-1 (PMC13457283; doi:10.1007/s40279-026-02428-1)
Supplement: Supplementary file 3 [file 40279_2026_2428_MOESM1_ESM.docx]

**Online Supplemental Material 10:** Network Meta-Regression Results

**Table 1:** Results from the strength network in the initial and sensitivity analyses, as well as the network meta-regressions performed with each covariate. A positive relative represents a relative effect in favour of the resistance training method in the treatment column.

| **Treatment** | **Comparator** | **Initial** | **Sensitivity** | **Age** | **Sex** | **Duration** | **Frequency** | **Outcome Region** | **Volume Load** | **Training to Failure** |
| --- | --- | --- | --- | --- | --- | --- | --- | --- | --- | --- |
| ACC | AEL | 0.06 (-0.73 - 0.86) | -0.12 (-0.48 - 0.25) | 0.14 (-0.72 - 1.01) | 0.15 (-0.66 - 0.97) | 0.07 (-0.78 - 0.91) | -0.03 (-0.66 - 0.61) | -0.03 (-0.95 - 0.87) | 0.04 (-0.98 - 1.01) | -0.11 (-2.76 - 1.78) |
| ACC | DRO | 0.33 (-0.51 - 1.2) | 0.16 (-0.23 - 0.54) | 0.25 (-0.64 - 1.14) | 0.39 (-0.76 - 1.62) | 0.35 (-0.51 - 1.25) | -0.11 (-0.88 - 0.65) | 0.2 (-0.76 - 1.13) | 0.37 (-0.63 - 1.38) | -0.02 (-0.81 - 0.77) |
| ACC | FLY | 0.35 (-0.34 - 1.04) | 0.03 (-0.3 - 0.35) | 0.34 (-0.36 - 1.04) | 0.38 (-0.27 - 1.03) | 0.35 (-0.43 - 1.14) | -0.17 (-0.77 - 0.41) | 0.27 (-0.54 - 1.06) | 0.33 (-0.44 - 1.08) | -0.23 (-3.16 - 2.6) |
| ACC | PTC | 0.17 (-0.46 - 0.79) | **-0.3**  **(-0.6 - 0)** | 0.15 (-0.49 - 0.8) | 0.33 (-0.31 - 0.94) | 0.11 (-0.57 - 0.8) | -0.21 (-0.64 - 0.21) | 0.09 (-0.66 - 0.83) | 0.17 (-0.5 - 0.84) | -0.24 (-2.59 - 1.7) |
| ACC | RRS | -0.13 (-0.76 - 0.48) | 0.08 (-0.23 - 0.39) | -0.22 (-0.86 - 0.43) | -0.19 (-0.79 - 0.41) | -0.19 (-0.86 - 0.48) | -0.34 (-1.16 - 0.5) | -0.26 (-1.02 - 0.48) | -0.15 (-0.83 - 0.53) | -0.18 (-1.23 - 0.9) |
| ACC | TRA | 0.18 (-0.29 - 0.64) | 0.01 (-0.22 - 0.23) | 0.15 (-0.35 - 0.66) | 0.19 (-0.28 - 0.67) | 0.18 (-0.33 - 0.69) | -0.37 (-1.03 - 0.29) | 0.1 (-0.53 - 0.7) | 0.19 (-0.33 - 0.71) | -0.5 (-1.88 - 0.81) |
| AEL | ACC | -0.06 (-0.86 - 0.73) | 0.12 (-0.25 - 0.48) | -0.14 (-1.01 - 0.72) | -0.15 (-0.97 - 0.66) | -0.07 (-0.91 - 0.78) | 0.03 (-0.61 - 0.66) | 0.03 (-0.87 - 0.95) | -0.04 (-1.01 - 0.98) | 0.11 (-1.78 - 2.76) |
| AEL | DRO | 0.28 (-0.69 - 1.23) | 0.27 (-0.15 - 0.7) | 0.11 (-0.94 - 1.12) | 0.23 (-0.96 - 1.46) | 0.29 (-0.69 - 1.27) | -0.08 (-0.88 - 0.71) | 0.23 (-0.78 - 1.23) | 0.33 (-0.87 - 1.62) | 0.09 (-1.77 - 2.67) |
| AEL | FLY | 0.29 (-0.52 - 1.11) | 0.14 (-0.23 - 0.52) | 0.2 (-0.67 - 1.07) | 0.23 (-0.56 - 1) | 0.28 (-0.61 - 1.18) | -0.15 (-0.78 - 0.47) | 0.3 (-0.57 - 1.15) | 0.28 (-0.71 - 1.29) | -0.12 (-1.96 - 2.32) |
| AEL | PTC | 0.11 (-0.64 - 0.86) | -0.18 (-0.54 - 0.17) | 0.01 (-0.81 - 0.84) | 0.17 (-0.56 - 0.92) | 0.05 (-0.79 - 0.85) | -0.18 (-0.64 - 0.29) | 0.11 (-0.69 - 0.93) | 0.12 (-0.81 - 1.13) | -0.13 (-2.7 - 2.97) |
| AEL | RRS | -0.19 (-0.96 - 0.57) | 0.2 (-0.16 - 0.56) | -0.35 (-1.2 - 0.47) | -0.34 (-1.09 - 0.39) | -0.26 (-1.08 - 0.54) | -0.31 (-1.15 - 0.54) | -0.23 (-1.04 - 0.58) | -0.19 (-1.15 - 0.82) | -0.07 (-2.05 - 2.63) |
| AEL | TRA | 0.12 (-0.53 - 0.75) | 0.13 (-0.16 - 0.42) | 0.01 (-0.71 - 0.72) | 0.04 (-0.6 - 0.68) | 0.11 (-0.58 - 0.79) | -0.34 (-1.03 - 0.34) | 0.12 (-0.56 - 0.82) | 0.14 (-0.68 - 1.07) | -0.39 (-2.46 - 2.18) |
| DRO | ACC | -0.33 (-1.2 - 0.51) | -0.16 (-0.54 - 0.23) | -0.25 (-1.14 - 0.64) | -0.39 (-1.62 - 0.76) | -0.35 (-1.25 - 0.51) | 0.11 (-0.65 - 0.88) | -0.2 (-1.13 - 0.76) | -0.37 (-1.38 - 0.63) | 0.02 (-0.77 - 0.81) |
| DRO | AEL | -0.28 (-1.23 - 0.69) | -0.27 (-0.7 - 0.15) | -0.11 (-1.12 - 0.94) | -0.23 (-1.46 - 0.96) | -0.29 (-1.27 - 0.69) | 0.08 (-0.71 - 0.88) | -0.23 (-1.23 - 0.78) | -0.33 (-1.62 - 0.87) | -0.09 (-2.67 - 1.77) |
| DRO | FLY | 0.01 (-0.86 - 0.89) | -0.13 (-0.52 - 0.26) | 0.09 (-0.79 - 0.97) | -0.01 (-1.22 - 1.13) | -0.01 (-0.95 - 0.92) | -0.07 (-0.82 - 0.69) | 0.07 (-0.83 - 0.98) | -0.04 (-1.1 - 0.99) | -0.21 (-3.09 - 2.66) |
| DRO | PTC | -0.17 (-0.99 - 0.66) | -0.46 (-0.83 - -0.09) | -0.1 (-0.95 - 0.73) | -0.06 (-1.24 - 1.07) | -0.24 (-1.11 - 0.61) | -0.1 (-0.73 - 0.55) | -0.11 (-0.98 - 0.77) | -0.2 (-1.14 - 0.72) | -0.22 (-2.49 - 1.5) |
| DRO | RRS | -0.47 (-1.3 - 0.35) | -0.07 (-0.45 - 0.3) | -0.47 (-1.31 - 0.37) | -0.58 (-1.79 - 0.53) | -0.54 (-1.41 - 0.29) | -0.23 (-1.18 - 0.73) | -0.46 (-1.32 - 0.4) | -0.52 (-1.44 - 0.39) | -0.16 (-0.85 - 0.55) |
| DRO | TRA | -0.16 (-0.88 - 0.56) | -0.15 (-0.46 - 0.16) | -0.1 (-0.82 - 0.62) | -0.19 (-1.35 - 0.84) | -0.18 (-0.92 - 0.54) | -0.26 (-1.06 - 0.55) | -0.1 (-0.86 - 0.66) | -0.18 (-1.02 - 0.63) | -0.48 (-1.79 - 0.69) |
| FLY | ACC | -0.35 (-1.04 - 0.34) | -0.03 (-0.35 - 0.3) | -0.34 (-1.04 - 0.36) | -0.38 (-1.03 - 0.27) | -0.35 (-1.14 - 0.43) | 0.17 (-0.41 - 0.77) | -0.27 (-1.06 - 0.54) | -0.33 (-1.08 - 0.44) | 0.23 (-2.6 - 3.16) |
| FLY | AEL | -0.29 (-1.11 - 0.52) | -0.14 (-0.52 - 0.23) | -0.2 (-1.07 - 0.67) | -0.23 (-1 - 0.56) | -0.28 (-1.18 - 0.61) | 0.15 (-0.47 - 0.78) | -0.3 (-1.15 - 0.57) | -0.28 (-1.29 - 0.71) | 0.12 (-2.32 - 1.96) |
| FLY | DRO | -0.01 (-0.89 - 0.86) | 0.13 (-0.26 - 0.52) | -0.09 (-0.97 - 0.79) | 0.01 (-1.13 - 1.22) | 0.01 (-0.92 - 0.95) | 0.07 (-0.69 - 0.82) | -0.07 (-0.98 - 0.83) | 0.04 (-0.99 - 1.1) | 0.21 (-2.66 - 3.09) |
| FLY | PTC | -0.18 (-0.82 - 0.46) | **-0.33 (-0.64 - -0.02)** | -0.19 (-0.84 - 0.46) | -0.05 (-0.66 - 0.53) | -0.23 (-0.94 - 0.46) | -0.03 (-0.43 - 0.39) | -0.18 (-0.83 - 0.47) | -0.16 (-0.88 - 0.57) | -0.01 (-2.6 - 3.46) |
| FLY | RRS | -0.48 (-1.13 - 0.16) | 0.06 (-0.26 - 0.37) | -0.56 (-1.22 - 0.1) | -0.57 (-1.14 - 0.01) | -0.54 (-1.26 - 0.16) | -0.16 (-0.98 - 0.67) | -0.53 (-1.2 - 0.14) | -0.48 (-1.19 - 0.25) | 0.05 (-2.86 - 3.01) |
| FLY | TRA | -0.17 (-0.68 - 0.33) | -0.02 (-0.25 - 0.22) | -0.19 (-0.7 - 0.31) | -0.19 (-0.64 - 0.25) | -0.17 (-0.75 - 0.39) | -0.19 (-0.85 - 0.44) | -0.17 (-0.67 - 0.34) | -0.14 (-0.7 - 0.45) | -0.27 (-3 - 2.57) |
| PTC | ACC | -0.17 (-0.79 - 0.46) | **0.3**  **(0 - 0.6)** | -0.15 (-0.8 - 0.49) | -0.33 (-0.94 - 0.31) | -0.11 (-0.8 - 0.57) | 0.21 (-0.21 - 0.64) | -0.09 (-0.83 - 0.66) | -0.17 (-0.84 - 0.5) | 0.24 (-1.7 - 2.59) |
| PTC | AEL | -0.11 (-0.86 - 0.64) | 0.18 (-0.17 - 0.54) | -0.01 (-0.84 - 0.81) | -0.17 (-0.92 - 0.56) | -0.05 (-0.85 - 0.79) | 0.18 (-0.29 - 0.64) | -0.11 (-0.93 - 0.69) | -0.12 (-1.13 - 0.81) | 0.13 (-2.97 - 2.7) |
| PTC | DRO | 0.17 (-0.66 - 0.99) | **0.46 (0.09 - 0.83)** | 0.1 (-0.73 - 0.95) | 0.06 (-1.07 - 1.24) | 0.24 (-0.61 - 1.11) | 0.1 (-0.55 - 0.73) | 0.11 (-0.77 - 0.98) | 0.2 (-0.72 - 1.14) | 0.22 (-1.5 - 2.49) |
| PTC | FLY | 0.18 (-0.46 - 0.82) | **0.33 (0.02 - 0.64)** | 0.19 (-0.46 - 0.84) | 0.05 (-0.53 - 0.66) | 0.23 (-0.46 - 0.94) | 0.03 (-0.39 - 0.43) | 0.18 (-0.47 - 0.83) | 0.16 (-0.57 - 0.88) | 0.01 (-3.46 - 2.6) |
| PTC | RRS | -0.3 (-0.87 - 0.28) | **0.38 (0.1 - 0.68)** | -0.37 (-0.96 - 0.21) | -0.52 (-1.04 - 0.03) | -0.3 (-0.92 - 0.3) | -0.13 (-0.83 - 0.59) | -0.35 (-0.95 - 0.26) | -0.32 (-0.93 - 0.28) | 0.06 (-1.85 - 2.42) |
| PTC | TRA | 0.01 (-0.4 - 0.42) | **0.31 (0.11 - 0.51)** | 0 (-0.41 - 0.41) | -0.14 (-0.52 - 0.26) | 0.06 (-0.38 - 0.51) | -0.16 (-0.67 - 0.34) | 0.01 (-0.41 - 0.43) | 0.02 (-0.41 - 0.44) | -0.26 (-2.34 - 1.96) |
| RRS | ACC | 0.13 (-0.48 - 0.76) | -0.08 (-0.39 - 0.23) | 0.22 (-0.43 - 0.86) | 0.19 (-0.41 - 0.79) | 0.19 (-0.48 - 0.86) | 0.34 (-0.5 - 1.16) | 0.26 (-0.48 - 1.02) | 0.15 (-0.53 - 0.83) | 0.18 (-0.9 - 1.23) |
| RRS | AEL | 0.19 (-0.57 - 0.96) | -0.2 (-0.56 - 0.16) | 0.35 (-0.47 - 1.2) | 0.34 (-0.39 - 1.09) | 0.26 (-0.54 - 1.08) | 0.31 (-0.54 - 1.15) | 0.23 (-0.58 - 1.04) | 0.19 (-0.82 - 1.15) | 0.07 (-2.63 - 2.05) |
| RRS | DRO | 0.47 (-0.35 - 1.3) | 0.07 (-0.3 - 0.45) | 0.47 (-0.37 - 1.31) | 0.58 (-0.53 - 1.79) | 0.54 (-0.29 - 1.41) | 0.23 (-0.73 - 1.18) | 0.46 (-0.4 - 1.32) | 0.52 (-0.39 - 1.44) | 0.16 (-0.55 - 0.85) |
| RRS | FLY | 0.48 (-0.16 - 1.13) | -0.06 (-0.37 - 0.26) | 0.56 (-0.1 - 1.22) | 0.57 (-0.01 - 1.14) | 0.54 (-0.16 - 1.26) | 0.16 (-0.67 - 0.98) | 0.53 (-0.14 - 1.2) | 0.48 (-0.25 - 1.19) | -0.05 (-3.01 - 2.86) |
| RRS | PTC | 0.3 (-0.28 - 0.87) | **-0.38 (-0.68 - -0.1)** | 0.37 (-0.21 - 0.96) | 0.52 (-0.03 - 1.04) | 0.3 (-0.3 - 0.92) | 0.13 (-0.59 - 0.83) | 0.35 (-0.26 - 0.95) | 0.32 (-0.28 - 0.93) | -0.06 (-2.42 - 1.85) |
| RRS | TRA | 0.31 (-0.1 - 0.72) | -0.07 (-0.29 - 0.14) | 0.37 (-0.04 - 0.79) | **0.38 (0.02 - 0.74)** | 0.37 (-0.06 - 0.81) | -0.03 (-0.9 - 0.84) | 0.36 (-0.09 - 0.8) | 0.34 (-0.09 - 0.77) | -0.32 (-1.81 - 1.08) |

***Note.*** Relative effects are presented as SMD (95% CrI), with a positive effect favouring the condition in the treatment column. Results in bold highlight a 95% probability that the favoured condition presents a greater relative effect in developing strength. *Abbreviations*: ACC, accommodating resistance; AEL, accentuated eccentric loading; DRO, dropsets; FLY, flywheel; PTC, potentiation complexes; RRS, rest redistribution schemes; TRA, traditional resistance training.

**Table 2:** Results from the power network in the initial and sensitivity analyses, as well as the network meta-regressions performed with each covariate. A positive relative represents a relative effect in favour of the resistance training method in the treatment column.

| **Treatment** | **Comparator** | **Initial** | **Sensitivity** | **Age** | **Duration** | **Frequency** | **Outcome Region** | **Volume Load** | **Training to Failure** |
| --- | --- | --- | --- | --- | --- | --- | --- | --- | --- |
| PTC | RRS | 0.05 (-0.42 - 0.54) | -0.05 (-0.45 - 0.34) | -0.04 (-0.51 - 0.42) | 1.2 (-4.34 - 9.84) | -0.04 (-0.3 - 0.21) | -0.08 (-0.36 - 0.2) | -0.15 (-0.53 - 0.21) | 0.03 (-1.12 - 1.2) |
| PTC | TRA | 0.22 (-0.17 - 0.63) | 0.04 (-0.3 - 0.38) | 0.04 (-0.38 - 0.45) | 1.29 (-4.26 - 9.95) | 0.03 (-0.19 - 0.26) | -0.13 (-0.27 - 0.02) | 0.04 (-0.26 - 0.34) | 0.13 (-1 - 1.28) |
| RRS | PTC | -0.05 (-0.54 - 0.42) | 0.05 (-0.34 - 0.45) | 0.04 (-0.42 - 0.51) | -1.2 (-9.84 - 4.34) | 0.04 (-0.21 - 0.3) | 0.08 (-0.2 - 0.36) | 0.15 (-0.21 - 0.53) | -0.03 (-1.2 - 1.12) |
| RRS | TRA | 0.17 (-0.09 - 0.43) | 0.09 (-0.1 - 0.3) | 0.08 (-0.13 - 0.29) | 0.09 (-0.12 - 0.31) | 0.08 (-0.04 - 0.2) | -0.04 (-0.29 - 0.19) | 0.2 (-0.02 - 0.42) | 0.1 (-0.13 - 0.32) |
| TRA | PTC | -0.22 (-0.63 - 0.17) | -0.04 (-0.38 - 0.3) | -0.04 (-0.45 - 0.38) | -1.29 (-9.95 - 4.26) | -0.03 (-0.26 - 0.19) | 0.13 (-0.02 - 0.27) | -0.04 (-0.34 - 0.26) | -0.13 (-1.28 - 1) |
| TRA | RRS | -0.17 (-0.43 - 0.09) | -0.09 (-0.3 - 0.1) | -0.08 (-0.29 - 0.13) | -0.09 (-0.31 - 0.12) | -0.08 (-0.2 - 0.04) | 0.04 (-0.19 - 0.29) | -0.2 (-0.42 - 0.02) | -0.1 (-0.32 - 0.13) |

***Note.*** Relative effects are presented as SMD (95% CrI), with a positive effect favouring the condition in the treatment column. Results in bold highlight a 95% probability that the favoured condition presents a greater relative effect in developing power. *Abbreviations*: PTC, potentiation complexes; RRS, rest redistribution schemes; TRA, traditional resistance training.

**Table 3:** Results from the jump network in the initial and sensitivity analyses, as well as the network meta-regressions performed with each covariate. A positive relative represents a relative effect in favour of the resistance training method in the treatment column.

| **Treatment** | **Comparator** | **Initial** | **Sensitivity** | **Age** | **Sex** | **Duration** | **Frequency** | **Volume Load** | **Training to Failure** |
| --- | --- | --- | --- | --- | --- | --- | --- | --- | --- |
| FLY | PTC | 0.35 (-0.07 - 0.78) | **0.28 (0.03 - 0.53)** | **0.29 (0.02 - 0.57)** | **0.29 (0.04 - 0.55)** | 0.27 (-0.01 - 0.56) | -0.16 (-0.49 - 0.18) | 0.23 (-0.15 - 0.6) | -0.04 (-1.33 - 1.52) |
| FLY | RRS | 0.02 (-0.46 - 0.52) | 0.17 (-0.15 - 0.48) | 0.18 (-0.15 - 0.51) | 0.17 (-0.14 - 0.49) | 0.16 (-0.17 - 0.5) | **-0.21 (-0.42 - 0)** | 0.2 (-0.7 - 1.08) | -0.15 (-0.62 - 0.3) |
| FLY | TRA | **0.36 (0.04 - 0.68)** | **0.21 (0.01 - 0.4)** | **0.22 (0 - 0.44)** | **0.22 (0.02 - 0.41)** | 0.21 (-0.01 - 0.42) | **-0.28 (-0.55 - -0.01)** | 0.15 (-0.18 - 0.47) | -0.21 (-1.74 - 1.5) |
| PTC | FLY | -0.35 (-0.78 - 0.07) | **-0.28 (-0.53 - -0.03)** | **-0.29 (-0.57 - -0.02)** | **-0.29 (-0.55 - -0.04)** | -0.27 (-0.56 - 0.01) | 0.16 (-0.18 - 0.49) | -0.23 (-0.6 - 0.15) | 0.04 (-1.52 - 1.33) |
| PTC | RRS | -0.33 (-0.8 - 0.13) | -0.11 (-0.41 - 0.18) | -0.11 (-0.42 - 0.19) | -0.12 (-0.41 - 0.17) | -0.11 (-0.43 - 0.21) | -0.05 (-0.32 - 0.21) | -0.03 (-0.88 - 0.85) | -0.11 (-1.76 - 1.12) |
| PTC | TRA | 0.01 (-0.27 - 0.28) | -0.07 (-0.23 - 0.09) | -0.07 (-0.23 - 0.09) | -0.07 (-0.24 - 0.08) | -0.06 (-0.24 - 0.11) | -0.12 (-0.44 - 0.2) | -0.08 (-0.26 - 0.1) | -0.17 (-1.63 - 1.34) |
| RRS | FLY | -0.02 (-0.52 - 0.46) | -0.17 (-0.48 - 0.15) | -0.18 (-0.51 - 0.15) | -0.17 (-0.49 - 0.14) | -0.16 (-0.5 - 0.17) | **0.21 (0 - 0.42)** | -0.2 (-1.08 - 0.7) | 0.15 (-0.3 - 0.62) |
| RRS | PTC | 0.33 (-0.13 - 0.8) | **0.11 (-0.18 - 0.41)** | 0.11 (-0.19 - 0.42) | 0.12 (-0.17 - 0.41) | 0.11 (-0.21 - 0.43) | 0.05 (-0.21 - 0.32) | 0.03 (-0.85 - 0.88) | 0.11 (-1.12 - 1.76) |
| RRS | TRA | 0.34 (-0.04 - 0.71) | 0.05 (-0.19 - 0.29) | 0.04 (-0.21 - 0.3) | 0.04 (-0.2 - 0.28) | 0.05 (-0.22 - 0.31) | -0.07 (-0.24 - 0.1) | -0.04 (-0.92 - 0.79) | -0.06 (-1.58 - 1.61) |
| TRA | FLY | **-0.36 (-0.68 - -0.04)** | **-0.21 (-0.4 - -0.01)** | **-0.22 (-0.44 - 0)** | **-0.22 (-0.41 - -0.02)** | -0.21 (-0.42 - 0.01) | **0.28 (0.01 - 0.55)** | -0.15 (-0.47 - 0.18) | 0.21 (-1.5 - 1.74) |
| TRA | PTC | -0.01 (-0.28 - 0.27) | 0.07 (-0.09 - 0.23) | 0.07 (-0.09 - 0.23) | 0.07 (-0.08 - 0.24) | 0.06 (-0.11 - 0.24) | 0.12 (-0.2 - 0.44) | 0.08 (-0.1 - 0.26) | 0.17 (-1.34 - 1.63) |
| TRA | RRS | -0.34 (-0.71 - 0.04) | -0.05 (-0.29 - 0.19) | -0.04 (-0.3 - 0.21) | -0.04 (-0.28 - 0.2) | -0.05 (-0.31 - 0.22) | 0.07 (-0.1 - 0.24) | 0.04 (-0.79 - 0.92) | 0.06 (-1.61 - 1.58) |

***Note.*** Relative effects are presented as SMD (95% CrI), with a positive effect favouring the condition in the treatment column. Results in bold highlight a 95% probability that the favoured condition presents a greater relative effect in developing jump performance. *Abbreviations*: FLY, flywheel; PTC, potentiation complexes; RRS, rest redistribution schemes; TRA, traditional resistance training.
