# Supplementary material for: The Effects of Advanced Resistance Training Prescription Methods on Strength, Power, Hypertrophy, and Performance Adaptations in Healthy Adults: A Systematic Review and Bayesian Network Meta-analysis
Source: Sports Med. 2026 Apr 8;56(8):1955–77. doi: 10.1007/s40279-026-02428-1 (PMC13457283; doi:10.1007/s40279-026-02428-1)
Supplement: Supplementary file 4 [file 40279_2026_2428_MOESM2_ESM.docx]

**Online Supplemental Material 11:** Network Meta-Regression Model Comparisons

**Fig. 1:** Strength network meta-regression model comparisons for the initial analysis and the model for each covariate. *Abbreviations*: pD, number of effective parameters; Dres, residual deviance; DIC, deviance information criteria; Wik, contribution of individual study arms to residual deviance; leverageik, leverage (high leverage suggests larger influence of a study arm on the model estimates).

**Fig. 2:** Power network meta-regression model comparisons for the initial analysis and the model for each covariate. Abbreviations: pD, number of effective parameters; Dres, residual deviance; DIC, deviance information criteria; Wik, contribution of individual study arms to residual deviance; leverageik, leverage (high leverage suggests larger influence of a study arm on the model estimates).

**Fig. 3:** Jump network meta-regression model comparisons for the initial analysis and the model for each covariate. Abbreviations: pD, number of effective parameters; Dres, residual deviance; DIC, deviance information criteria; Wik, contribution of individual study arms to residual deviance; leverageik, leverage (high leverage suggests larger influence of a study arm on the model estimates).
