# Supplementary material for: The Effects of Advanced Resistance Training Prescription Methods on Strength, Power, Hypertrophy, and Performance Adaptations in Healthy Adults: A Systematic Review and Bayesian Network Meta-analysis
Source: Sports Med. 2026 Apr 8;56(8):1955–77. doi: 10.1007/s40279-026-02428-1 (PMC13457283; doi:10.1007/s40279-026-02428-1)
Supplement: Supplementary file 5 [file 40279_2026_2428_MOESM3_ESM.docx]

**Online Supplemental Material 1:** Search Strategies

EBSCO Database (SPORTDiscus, CINAHL, MEDLINE):

( adult* OR athlete* ) AND ( train* OR exercis* OR resistance N1 (train* exercis*) OR weight N1 (train* OR exercis* OR -lifting) OR weightlifting OR strength N1 (train* OR exercis*) ) AND ( complex N1 (train* OR set* OR structure*) OR contrast N1 (train* OR set* OR structure*) OR resistance N1 (variable OR accommodating) OR band* OR chain* OR eccentric N2 (accentuated OR overload) OR flywheel OR inertia OR cluster OR "rest redistribution" OR superset OR dropset OR "paired sets" OR "drop set" OR "descending set") AND ( velocity OR torque OR strength OR power OR muscle N1 (hypertrophy OR growth OR thickness) OR muscular N1 (hypertrophy OR growth OR thickness) OR force OR jump OR sprint OR speed) NOT (injur* OR nursing OR tendinopathy OR cancer OR rat OR mice OR elderly OR child*)

Scopus:

(TITLE-ABS-KEY(adult OR athlete)) AND (TITLE-ABS-KEY(train* OR exercis* OR (resistance W/1 (train* OR exercis*)) OR (weight W/1 (train* OR exercis* OR lifting)) OR (weightlifting W/1 strength W/1 (train* OR exercis*)))) AND (TITLE-ABS-KEY((complex W/1 (train* OR set* OR structure*)) OR (contrast W/1 (train* OR set* OR structure*)) OR (resistance W/1 (variable OR accommodating)) OR band* OR chain* OR (eccentric W/2 (accentuated OR overload)) OR flywheel OR inertia OR cluster OR "rest redistribution" OR superset OR dropset OR "drop set" OR "paired sets" OR "descending set")) AND (TITLE-ABS-KEY(velocity OR torque OR strength OR power OR (muscle W/1 (hypertrophy OR growth OR thickness)) OR (muscular W/1 (hypertrophy OR growth OR thickness)) OR force OR jump OR sprint OR speed)) AND NOT (TITLE-ABS-KEY(Injur* OR Nursing OR tendinopathy OR Cancer OR rat OR mice OR Elderly OR child*))

PubMed:

(((((adult[Title/Abstract] OR athlete[Title/Abstract])) AND ((train*[Title/Abstract] OR exercis*[Title/Abstract] OR ("resistance training"[Title/Abstract] OR "resistance exercise"[Title/Abstract]) OR ("weight training"[Title/Abstract] OR "weight exercise"[Title/Abstract] OR "weight lifting"[Title/Abstract]) OR "weightlifting"[Title/Abstract]))) AND ((("complex training"[Title/Abstract] OR "complex set"[Title/Abstract] OR "complex structure"[Title/Abstract]) OR ("contrast training"[Title/Abstract] OR "contrast set"[Title/Abstract] OR "contrast structure"[Title/Abstract]) OR ("resistance variable"[Title/Abstract] OR "accommodating resistance"[Title/Abstract]) OR band*[Title/Abstract] OR chain*[Title/Abstract] OR ("eccentric accentuated"[Title/Abstract] OR "eccentric overload"[Title/Abstract]) OR flywheel[Title/Abstract] OR inertia[Title/Abstract] OR cluster[Title/Abstract] OR "rest redistribution"[Title/Abstract] OR superset[Title/Abstract] OR dropset[Title/Abstract] OR "drop set"[Title/Abstract] OR "paired sets"[Title/Abstract] OR "descending set"[Title/Abstract]))) AND ((velocity[Title/Abstract] OR torque[Title/Abstract] OR strength[Title/Abstract] OR power[Title/Abstract] OR ("muscle hypertrophy"[Title/Abstract] OR "muscle growth"[Title/Abstract] OR "muscle thickness"[Title/Abstract]) OR ("muscular hypertrophy"[Title/Abstract] OR "muscular growth"[Title/Abstract] OR "muscular thickness"[Title/Abstract]) OR force[Title/Abstract] OR jump[Title/Abstract] OR sprint[Title/Abstract] OR speed[Title/Abstract]))) NOT ((injur*[Title/Abstract] OR nursing[Title/Abstract] OR tendinopathy[Title/Abstract] OR cancer[Title/Abstract] OR rat[Title/Abstract] OR mice[Title/Abstract] OR elderly[Title/Abstract] OR child*[Title/Abstract]))
