# Supplementary material for: The Effects of Advanced Resistance Training Prescription Methods on Strength, Power, Hypertrophy, and Performance Adaptations in Healthy Adults: A Systematic Review and Bayesian Network Meta-analysis
Source: Sports Med. 2026 Apr 8;56(8):1955–77. doi: 10.1007/s40279-026-02428-1 (PMC13457283; doi:10.1007/s40279-026-02428-1)
Supplement: Supplementary file 6 [file 40279_2026_2428_MOESM4_ESM.docx]

**Online Supplemental Material 2:** Systematic Reviews Searched

[1-17]

1.	Sødal LK, Kristiansen E, Larsen S, van den Tillaar R. Effects of Drop Sets on Skeletal Muscle Hypertrophy: A Systematic Review and Meta-analysis. Sports Medicine - Open. 2023 2023/07/31;9(1):66. <https://doi.org/10.1186/s40798-023-00620-5>

2.	Tufano JJ, Brown LE, Haff GG. Theoretical and Practical Aspects of Different Cluster Set Structures: A Systematic Review. The Journal of Strength & Conditioning Research. 2017;31(3):848-67. <https://doi.org/10.1519/jsc.0000000000001581>

3.	Thapa R, Narvariya P, Weldon A, Talukdar K, Ramirez R. Can complex contrast training interventions improve aerobic endurance, maximal strength, and repeated sprint ability in soccer players?: A systematic review and meta-analysis. Montenegrin Journal of Sports Science and Medicine. 2022;11(2):3-13.

4.	Cormier P, Freitas TT, Seaman K. A systematic review of resistance training methodologies for the development of lower body concentric mean power, peak power, and mean propulsive power in team-sport athletes. Sports Biomechanics. 2024 2024/10/02;23(10):1229-62. <https://doi.org/10.1080/14763141.2021.1948601>

5.	Raya-González J, Prat-Luri A, López-Valenciano A, Sabido R, Hernández-Davó JL. Effects of Flywheel Resistance Training on Sport Actions. A Systematic Review and Meta-Analysis. Journal of Human Kinetics. 2021;77:191-204. <https://doi.org/10.2478/hukin-2021-0020>

6.	Allen WJC, De Keijzer KL, Raya-González J, Castillo D, Coratella G, Beato M. Chronic effects of flywheel training on physical capacities in soccer players: a systematic review. Research in Sports Medicine. 2023 2023/05/04;31(3):228-48. <https://doi.org/10.1080/15438627.2021.1958813>

7.	Maroto-Izquierdo S, García-López D, Fernandez-Gonzalo R, Moreira OC, González-Gallego J, de Paz JA. Skeletal muscle functional and structural adaptations after eccentric overload flywheel resistance training: a systematic review and meta-analysis. Journal of Science and Medicine in Sport. 2017 2017/10/01/;20(10):943-51. <https://doi.org/https://doi.org/10.1016/j.jsams.2017.03.004>

8.	Shi L, Cai Z, Chen S, Han D. Acute effects of variable resistance training on force, velocity, and power measures: a systematic review and meta-analysis. PeerJ. 2022 2022/08/17;10:e13870. <https://doi.org/10.7717/peerj.13870>

9.	Thapa RK, Lum D, Moran J, Ramirez-Campillo R. Effects of complex training on sprint, jump, and change of direction ability of soccer players: A systematic review and meta-analysis. Frontiers in Psychology. 2021;11:627869.

10.	Lin Y, Xu Y, Hong F, Li J, Ye W, Korivi M. Effects of Variable-Resistance Training Versus Constant-Resistance Training on Maximum Strength: A Systematic Review and Meta-Analysis. International Journal of Environmental Research and Public Health. 2022;19(14):8559.

11.	Lesinski M, Mühlbauer T, Büsch D, Granacher U. Effects of complex training on strength and speed performance in athletes: a systematic review. Effects of complex training on athletic performance. Sportverletzung Sportschaden: Organ der Gesellschaft fur Orthopadisch-Traumatologische Sportmedizin. 2014;28(2):85-107.

12.	de Oliveira PA, Blasczyk JC, Junior GS, Lagoa KF, Soares M, de Oliveira RJ, et al. Effects of Elastic Resistance Exercise on Muscle Strength and Functional Performance in Healthy Adults: A Systematic Review and Meta-Analysis. Journal of Physical Activity and Health. 2017 01 Apr. 2017;14(4):317-27. <https://doi.org/10.1123/jpah.2016-0415>

13.	Chaabene H, Markov A, Prieske O, Moran J, Behrens M, Negra Y, et al. Effect of Flywheel versus Traditional Resistance Training on Change of Direction Performance in Male Athletes: A Systematic Review with Meta-Analysis. International Journal of Environmental Research and Public Health. 2022;19(12):7061.

14.	Andersen V, Prieske O, Stien N, Cumming K, Solstad TEJ, Paulsen G, et al. Comparing the effects of variable and traditional resistance training on maximal strength and muscle power in healthy adults: A systematic review and meta-analysis. Journal of Science and Medicine in Sport. 2022 2022/12/01/;25(12):1023-32. <https://doi.org/https://doi.org/10.1016/j.jsams.2022.08.009>

15.	Cormier P, Freitas TT, Rubio-Arias JÁ, Alcaraz PE. Complex and Contrast Training: Does Strength and Power Training Sequence Affect Performance-Based Adaptations in Team Sports? A Systematic Review and Meta-analysis. The Journal of Strength & Conditioning Research. 2020;34(5):1461-79. <https://doi.org/10.1519/jsc.0000000000003493>

16.	Jukic I, Ramos AG, Helms ER, McGuigan MR, Tufano JJ. Acute Effects of Cluster and Rest Redistribution Set Structures on Mechanical, Metabolic, and Perceptual Fatigue During and After Resistance Training: A Systematic Review and Meta-analysis. Sports Medicine. 2020 2020/12/01;50(12):2209-36. <https://doi.org/10.1007/s40279-020-01344-2>

17.	Krzysztofik M, Wilk M, Wojdała G, Gołaś A. Maximizing Muscle Hypertrophy: A Systematic Review of Advanced Resistance Training Techniques and Methods. International Journal of Environmental Research and Public Health. 2019;16(24):4897.
