# Supplementary material for: The Effects of Advanced Resistance Training Prescription Methods on Strength, Power, Hypertrophy, and Performance Adaptations in Healthy Adults: A Systematic Review and Bayesian Network Meta-analysis
Source: Sports Med. 2026 Apr 8;56(8):1955–77. doi: 10.1007/s40279-026-02428-1 (PMC13457283; doi:10.1007/s40279-026-02428-1)
Supplement: Supplementary file 7 [file 40279_2026_2428_MOESM5_ESM.docx]

**Online Supplemental Material 3:** Data extracted from each study

Study Details:

- Study Title
- Lead Author
- Year
- Study Design
- Training Conditions

Participant Characteristics:

- Sample size
- Age
- Height
- Weight
- Sex
- Percentage of male participants
- Population
- Resistance training experience
- Relative strength (inc. exercise)

Intervention Characteristics:

- Intervention duration (weeks)
- Training frequency (sessions/week)
- Region of exercises performed
- Exercise performed (inc. auxiliary exercises)
- Number of exercises performed (total, lower, upper)
  - Lower/upper ratio
- Minimum load used
- Maximum load used
- Relative weekly volume load (total, lower, upper)
- Additional load (i.e., accommodating resistance and accentuated eccentric loading)
- Dropsets:
  - Dropset loads
- Potentiation Complexes:
  - Potentiation exercise, load, and rest duration
- Rest Redistribution Schemes
  - Rest time
  - Rest frequency
- Supersets:
  - Superset Pairs
  - Rest Time

Outcomes:

- Outcome category
- Outcome measure
- Outcome measure region
- Pre- and post-results (i.e., mean and standard deviation)
