# Supplementary material for: The Effects of Advanced Resistance Training Prescription Methods on Strength, Power, Hypertrophy, and Performance Adaptations in Healthy Adults: A Systematic Review and Bayesian Network Meta-analysis
Source: Sports Med. 2026 Apr 8;56(8):1955–77. doi: 10.1007/s40279-026-02428-1 (PMC13457283; doi:10.1007/s40279-026-02428-1)
Supplement: Supplementary file 8 [file 40279_2026_2428_MOESM6_ESM.docx]

**Online Supplemental Material 4:** Outcome hierarchy

Outcome hierarchy adapted from Currier et al. [1], in which the highest-ranked outcome (by order of appearance below) will be selected for analysis:

Things to add to hierarchy: Hypertrophy regional differentiation

Strength:

1. 1-Repetition Maximum
   a. Lower-Body
      i. Squat
      ii. Deadlift
      iii. Leg Press
      iv. Knee extension
   b. Upper-Body
      i. Bench/Chest Press
      ii. Prone Row
      iii. Bicep curl/Elbow Flexion
      iv. Elbow Extension
2. 2-6-Repetition Maximum
   a. Same as “1-Repetition Maximum”
3. Isokinetic
   a. Lower
      i. Knee extension (angular velocity closest to 60°/s) (right leg over left leg)
      ii. Knee flexion (angular velocity closest to 60°/s) (right leg over left leg)
   b. Upper
4. Isometric
   a. Lower
      i. Knee extension (angular velocity closest to 60°/s) (right leg over left leg)
      ii. Knee flexion (angular velocity closest to 60°/s) (right leg over left leg)
   b. Upper

Power:

1. Dynamic Exercise Peak Power Output
   a. Constant Load (e.g., load that elicits V1m/s; jump squat with 30kg)
   b. RM
2. Isokinetic Peak Power Output
3. Jump Peak Power Output
4. Wingate Peak Power Output
5. Dynamic Exercise Mean Power Output
   a. Constant Load (e.g., load that elicits V1m/s; jump squat with 30kg)
   b. RM
   c. Set Mean Power
6. Dynamic Exercise Mean Power Output
   a. Constant Load (e.g., load that elicits V1m/s; jump squat with 30kg)
   b. RM
7. Isokinetic Mean Power Output
   i. Knee extension (angular velocity closest to 60°/s) (right leg over left leg)
8. Jump Mean Power Output
9. Velocity Output with a Consistent External Load

Hypertrophy:

1. Magnetic Resonance Imaging (MRI)
   a. Muscle group volume (e.g., quadriceps)
      i. Lower-body
      ii. Upper-body
   b. Muscle volume
      i. Lower-body
         1. Vastus lateralis
         2. Rectus femoris
         3. Vastus medialis
      ii. Upper-body
          1. Pectoralis major
          2. Biceps brachii
          3. Triceps brachii
   c. Muscle group cross-sectional area (CSA)
      i. Lower-body
      ii. Upper-body
   d. Muscle CSA
      i. Lower-body
         1. Vastus lateralis
            a. Middle
            b. Distal
            c. Proximal
         2. Rectus femoris
            a. Middle
            b. Distal
            c. Proximal
         3. Vastus Medialis
            a. Middle
            b. Distal
            c. Proximal
   e. Muscle group thickness
      i. Lower-body
      ii. Upper-body
   f. Muscle thickness
      i. Lower-body
         1. Vastus lateralis
            a. Middle
            b. Distal
            c. Proximal
         2. Rectus femoris
            a. Middle
            b. Distal
            c. Proximal
         3. Vastus Medialis
            a. Middle
            b. Distal
            c. Proximal
2. Computed Tomography (CT):
   a. Same as “MRI”
3. Ultrasound:
   a. Muscle volume
      i. Lower-body
         1. Vastus lateralis
         2. Rectus femoris
         3. Vastus medialis
      ii. Upper-body
          1. Pectoralis major
          2. Biceps brachii
          3. Triceps brachii
   b. Muscle CSA
      i. Lower-body
         1. Vastus lateralis
            a. Middle
            b. Distal
            c. Proximal
         2. Rectus femoris
            a. Middle
            b. Distal
            c. Proximal
         3. Vastus Medialis
            a. Middle
            b. Distal
            c. Proximal
   c. Muscle thickness
      i. Lower-body
         1. Vastus lateralis
            a. Middle
            b. Distal
            c. Proximal
         2. Rectus femoris
            a. Middle
            b. Distal
            c. Proximal
         3. Vastus Medialis
            a. Middle
            b. Distal
            c. Proximal

Jump:

1. Vertical Jump Height
   a. CMJ
      i. Angle closest to 90 degrees
   b. SJ
   c. Drop/Depth Jump
   d. Single Leg CMJ
   e. Single Leg SJ
   f. “Vertical jump” or “Jump Test”
2. Vertical Jump Flight time
   a. Same as “vertical jump height”
3. Horizontal Jump Height
   a. Broad Jump
   b. Single-Leg Broad Jump`
   c. “Horizontal Jump”
4. Vertical Jump Velocity

Sprint:

1. Velocity
   a. 40m sprint
   b. 20m flying sprint
   c. 30m sprint
   d. 20m sprint
   e. 10m sprint
2. Time
   a. Same as “Velocity”

1.	Currier BS, Mcleod JC, Banfield L, Beyene J, Welton NJ, D'Souza AC, et al. Resistance training prescription for muscle strength and hypertrophy in healthy adults: a systematic review and Bayesian network meta-analysis. Br J Sports Med. 2023;57(18):1211-20. <https://doi.org/https://doi.org/10.1136/bjsports-2023-106807>
