# Supplementary material for: The Effects of Advanced Resistance Training Prescription Methods on Strength, Power, Hypertrophy, and Performance Adaptations in Healthy Adults: A Systematic Review and Bayesian Network Meta-analysis
Source: Sports Med. 2026 Apr 8;56(8):1955–77. doi: 10.1007/s40279-026-02428-1 (PMC13457283; doi:10.1007/s40279-026-02428-1)
Supplement: Supplementary file 9 [file 40279_2026_2428_MOESM7_ESM.docx]

**Online Supplemental Material 5:** Details of Included Studies

| Study | Resistance Training Methods | Sample | Intervention | Advanced Method Details | Outcomes |
| --- | --- | --- | --- | --- | --- |
| Alemdaroglu et al. 2013 [1] | TRA  PTC | n: TRA = 8 (3 female)  PTC = 8 (3 female)  Age: TRA = 21.6 ± 2.3  PTC = 21.3 ± 2.1  Population: recreationally trained | 6 weeks  2 sessions/week | PTC: Complex pairs (Leg curl + front tuck jump; Split squat + split jump; leg press + squat jump)  Intra-pair rest = 1 minutes | Strength: Isokinetic 60-degree knee extension  Jump: CMJ height |
| Andersen et al. 2015 [2] | TRA  ACC | n: TRA = 15 (15 female)  ACC = 15 (15 female)  Age: TRA = 24.0 ± 4.0  ACC = 24.0 ± 5.0  Population: recreationally trained | 10 weeks  2 sessions/week | ACC: Elastic bands (180 newtons) | Strength: 6RM squat  Jump: CMJ (90-degree knee angle) height |
| Angleri et al. 2017 [3] | TRA  DRO | n: TRA = 32 (0 female)  DRO = 16 (0 female)  Age = 27.0 ± 3.9  Population: recreationally trained | 12 weeks  2 sessions/week | DRO = 20% of load reduction x 2 | Strength: 1RM 45-degree leg press  Hypertrophy: Vastus lateralis CSA (ultrasound) |
| Arazi et al. 2014 [4] | TRA  PTC | n: TRA = 7 (7 female)  PTC = 7 (7 female)  Age: TRA = 20.71 ± 1.10  PTC = 20.57 ± 0.5  Population: untrained | 6 weeks  2 sessions/week | PTC: Complex pairs (half-squat + depth jump; knee extension + multiple jumps; knee flexion + zigzag drill; single leg lunge + lunge jump)  Intra-pair rest = 1 minutes | Strength: 1RM leg press  Jump: Vertical jump height  Sprint: 20-meter time |
| Arazi et al. 2018 [5] | TRA  RRS | n: TRA = 10 (10 female)  RRS = 10 (10 female)  Age: TRA = 18.7 ± 1.5  RRS = 18.2 ± 2.4  Population: volleyball athletes | 8 weeks  3 sessions/week | RRS: Cluster rest = 10-30 seconds  Cluster frequency = 2-3 | Strength: 1RM squat  Jump: CMJ height  Sprint: 20-meter time  Power: CMJ power |
| Arazi et al. 2020 [6] | TRA  ACC | n: TRA = 12 (12 female)  ACC = 12 (12 female)  Age: TRA = 23.58 ± 3.84  ACC = 23.75 ± 3.64  Population: untrained eumenorrheic | 8 weeks  3 sessions/week | ACC: Chains (15% total resistance) | Strength: 1RM squat |
| Arazi et al. 2021 [7] | TRA  RRS | n: TRA = 8 (0 female)  RRS = 8 (0 female)  Age: TRA = 22.3 ± 0.9  RRS = 23.6 ± 1.3  Population: powerlifters | 8 weeks  3 sessions/week | RRS: Cluster rest = 20-30 seconds  Cluster frequency = 2 | Strength: 1RM back squat |
| Arsenis et al. 2021 [8] | TRA  FLY | n: TRA = 16 (0 female)  FLY = 16 (0 female)  Age: TRA = 21.31 ± 1.25  FLY = 20.63 ± 1.45  Population: well-trained | 8 weeks  2 sessions/week | FLY: loading = 80-100% of optimal power load | Strength: 1RM semi squat  Jump: CMJ Height  Sprint: 30-meter time |
| Banks et al. 2024 [9] | TRA  FLY | n: TRA = 12 (7 female)  FLY = 11 (7 female)  Age: TRA = 25.75 ± 3.00  FLY = 22.73 ± 3.10 | 10 weeks  3 sessions/week | FLY: Inertial load = n/a | Strength: 1RM squat  Jump: CMJ height |
| Baruah et al. 2024 [10] | TRA  PTC | n: TRA = 8 (0 female)  PTC = 14 (0 female)  Age: TRA = 21.3 ± 2.1  TRA = 21.3 ± 1.8  Population: physically active | 7 weeks  2 sessions/week | PTC: Complex pairs (back squat + countermovement jumps; walking lunges + split jumps; romanian deadlifts + standing broad jumps; single standing calf raises + pogo jumps)  Intra-pair rest: n/a | Strength: 1RM Squat  Jump: CMJ height  Sprint: 30-metre time |
| Brandenburg & Docherty 2002 [11] | TRA AEL | n: TRA = 10 (0 female)  AEL = 8 (0 female)  Age: TRA = n/a  AEL = n/a  Population: active university-aged | 9 weeks  2-3 sessions/week | AEL: additional eccentric load = 30-45% 1RM | Strength: 1RM concentric elbow flexion  Hypertrophy: elbow flexor CSA (MRI) |
| Brito et al. 2014 [12] | TRA  PTC | n: TRA = 12 (0 female)  PTC = 12 (0 female)  Age: TRA = 20.3 ± 0.9  PTC = 19.9 ± 0.5  Population: soccer players | 9 weeks  2 sessions/week | PTC: Complex pairs (squat + high skipping/5m sprint; calf extension + vertical jump/ball headers; leg extension + vertical jump/drop jump)  Intra-pair rest = n/a | Strength: 1RM squat  Sprint: 20-metre time |
| Burnham et al. 2010 [13] | TRA  ACC | n: TRA = 9 (9 female)  ACC = 10 (10 female)  Age = 19.8 ± 1.6  Population: NCAA division II athletes | 8 weeks  2 sessions/week | ACC: Elastic bands (5% 1RM) | Strength: 1RM bench press |
| Caruso et al. 2008 [14] | TRA  FLY | n: TRA = 10 (3 female)  FLY = 9 (2 female)  Age = n/a  Population: college-aged | 6 weeks  2 sessions/week | FLY: Inertial load = n/a | Strength: Leg extension torque (3.14 rad^.^s)  Jump: Depth jump height  Power: Depth jump power |
| Cin et al. 2021 [15] | TRA  RRS | n: TRA = 14 (0 female)  RRS = 14 (0 female)  Age: TRA = 27.40 ± 1.37  RRS = 27.10 ± 1.60  Population: college-aged | 6 weeks  3 sessions/week | RRS: Cluster rest = 20 seconds  Cluster frequency = 3 | Strength: 1RM back squat  Jump: CMJ height  Sprint: 20-metre time |
| Cornejo-Daza et al. 2025 [16] | TRA  RRS | n: TRA = 10 (0 female)  RRS = 10 (0 female)  Age = 27.40 ± 1.37  Population: resistance-trained | 8 weeks  2 sessions/week | RRS: Cluster rest = 30 seconds  Cluster frequency = 3-5 | Strength: 1RM Squat  Jump: CMJ height  Hypertrophy: Vastus lateralis volume |
| Cuevas-Aburto et al. 2021 [17] | TRA  RRS | n: TRA = 12 (0 female)  RRS = 13 (0 female)  Age: TRA = 21.0 ± 2.5  RRS = 20.3 ± 3.1  Population: recreationally trained | 6 weeks  2 sessions/week | RRS: RRS rest = 31 seconds  RRS frequency = 30 | Strength: 1RM bench press  Power: Bench press throw power |
| Dai et al. 2025 [18] | TRA  ACC | n: TRA = 14 (n/a)  ACC = 14 (n/a)  Age: TRA = 20.10 ± 0.94  ACC = 20.3 ± 0.82  Population: college basketball athletes | 8 weeks  2 sessions/week | ACC: Elastic bands (30% 1RM of total load) | Strength: 1RM squat  Jump: CMJ height  Sprint: 10-metre time |
| Djuric et al. 2016 [19] | TRA  ACC | n: TRA = 12 (0 female)  ACC = 12 (0 female)  Age = 20.5 ± 2.0  Population: physically active | 8 weeks  3 sessions/week | ACC: Elastic bands (157.2 newtons) | Strength: Bench press throw force  Power: Bench press throw power |
| Douglas et al. 2018 [20] | TRA  AEL | n: TRA = 7 (0 female)  AEL = 7 (0 female)  Age: 19.4 ± 0.8  Population: rugby players | 8 weeks  3 sessions/week | AEL: additional eccentric load = 14-29% 1RM | Strength: 1RM back squat (relative)  Power: Inertial bike peak power (relative)  Jump: Drop jump flight time  Sprint: 40m sprint maximum velocity |
| Eler et al. 2024 [21] | TRA  RRS | n: TRA = 16 (16 female)  RRS = 16 (16 female)  Age: TRA = 26.20 ± 6.16  RRS = 24.21 ± 2.87  Population: handball players | 6 weeks  3 sessions/week | RRS: Cluster rest = 20 seconds  Cluster frequency = 2 | Strength: 1RM back squat  Jump: Vertical jump height  Sprint: 20-metre time |
| Enes et al. 2021 [22] | TRA  DRO | n: TRA = 9 (0 female)  RRS = 9 (0 female)  Age: TRA = 23.2 ± 2.6  RRS = 22.4 ± 3.3  Population: trained subjects | 8 weeks  2 sessions/week | DRO: 20% 1RM reduction | Strength: 1RM back squat  Hypertrophy: Vastus lateralis (middle) muscle thickness (ultrasound) |
| Filetti et al. 2023 [23] | TRA  FLY | n: TRA = 7 (0 female)  FLY = 7 (0 female)  Age = 18.0 ± 1.0  Population: soccer players | 6 weeks  3 sessions/week (last week 1 session) | FLY: inertial load = 0.0611 – 0.0811 kg^.^m^2^ | Hypertrophy: Dominant vastus lateralis (middle) CSA (MRI) |
| Fink et al. 2018 [24] | TRA  DRO | n: TRA = 8 (0 female)  DRO = 8 (0 female)  Age: TRA = 22.8 ± 3.9  DRO = 21.6 ± 1.9  Population: recreationally trained | 6 weeks  2 sessions/week | DRO: 2 x 20% 1RM reductions | Hypertrophy: Triceps CSA (MRI) |
| Godard et al. [25] | TRA  AEL | n: 28 (12 female)  TRA = 9  AEL = 9  Age: TRA = 21.1 ± 1.5  AEL = 22.0 ± 3.0  Population: untrained | 10 weeks  2 sessions/week | AEL: additional eccentric load = 40% 1RM | Strength: 1RM knee extension |
| Hansen et al. 2011 [26] | TRA  RRS | n: TRA = 9 (0 female)  RRS = 9 (0 female)  Age: TRA = 22.8 ± 3.9  RRS = 21.6 ± 1.9  Population: rugby players | 8 weeks  2 sessions/week | RRS: Cluster rest = 10-30 seconds  Cluster frequency = 1-3 | Strength: 1RM back squat  Power: Squat jump |
| Iversen et al. 2024 [27] | TRA  SUP | n: TRA = 13 (6 female)  SUP = 13 (7 female)  Age: TRA = 26  SUP = 28  Population: untrained | 10 weeks  2 sessions/week | SUP: superset pairs (bench press + seated rows, leg press + pull-down) | Strength: 1RM leg press |
| Janicijevic et al. 2023 [28] | TRA  RRS | n: TRA = 15 (0 female)  RRS = 15 (0 female)  Age: TRA = 20.6 ± 2.8  RRS = 20.9 ± 3.8  Population: recreationally trained | 6 weeks  2 sessions/week | RRS: RRS rest = 31 seconds  RRS frequency = 30 | Power: Bench press throw  Jump: CMJ velocity |
| Freitas et al. 2019 [29] | TRA  PTC | n: TRA = 9 (0 female)  PTC = 9 (0 female)  Age = 21.3 ± 4.3  Population: basketball players | 6 weeks  2 sessions/week | PTC: Complex pairs (half-squat + half-squat [OPL]; bench-press + bench press [OPL]; hip thrust + hip thrust [OPL])  Intra-pair rest = 2.5 minutes | Strength: 1RM half-squat  Jump: CMJ height  Power: CMJ peak power  Sprint: 10-metre time |
| Friedmann-Bette et al. 2010 [30] | TRA  AEL | n: TRA = 11 (0 female)  AEL = 14 (0 female)  Age: TRA = 24.5 ± 4.2  AEL = 24.3 ± 3.7  Population: trained athletes | 6 weeks  3 sessions/week | AEL: additional eccentric load = 72% 1RM | Strength: 1RM concentric leg extension  Jump: Squat jump height  Hypertrophy: Whole quadriceps CSA (MRI) |
| Garcia-Orea et al. 2022 [31] | TRA  SUP | n: TRA = 8 (0 female)  SUP = 9 (0 female)  Age = 23.9 ± 5.3  Population: moderately strength-trained | 6 weeks  2 sessions/week | SUP: superset pairs (squat + bench press) | Strength: 1RM back squat  Jump: CMJ height |
| Kashiani et al. 2020 [32] | TRA  ACC | n: TRA = 18 (0 female)  ACC = 18 (0 female)  Age = 21.50 ± 1.95  Population: untrained | 12 weeks  2 sessions/week | ACC: Chains (35% total resistance) | Strength: 1RM overhead press |
| Li et al. 2019 [33] | TRA  PTC | n: TRA = 9 (0 female)  PTC = 10 (0 female)  Age: TRA = 21.22 ± 1.48  PTC = 20.20 ± 1.03  Population: collegiate long-distance runners | 8 weeks  3 sessions/week | PTC: Complex pairs (back squat + drop jump; Bulgarian squat + single leg hop; Romanian deadlift + double leg hurdle hop)  Intra-pair rest = 4 minutes | Strength: 1RM squat  Jump: CMJ height  Power: CMJ peak power |
| Li et al. 2021 [34] | TRA PTC | n: TRA = 13 (4 female)  PTC = 13 (4 female)  Age: TRA = 31.2 ± 3.8  PTC = 32.2 ± 3.3  Population: recreational marathon runners | 6 weeks  3 sessions/week | PTC: Complex pairs (back squat + drop jump; split squat + single leg hop; lunge walking + double leg hurdle hop)  Intra-pair rest = 4 minutes | Strength: 1RM squat  Jump: CMJ height |
| Liu et al. 2022 [35] | TRA  PTC | n: TRA = 15 (0 female)  PTC = 15 (0 female)  Age: TRA = 23.9 ± 3.9  PTC = 22.7 ± 1.9  Population: professional firefighters | 12 weeks  2 sessions/week | PTC: Complex pairs (squat + squat jump; barbell bench press + high-five push-ups; deadlift + high pull; weight-bearing lunge + split-leg squat jump; dumbbell bench press + kneeling forward medicine ball; military press + push press; reverse grip loaded pull-ups + elastic band pull-up)  Intra-pair rest = 4 minutes | Strength: 1RM back squat  Jump: CMJ height |
| Lundberg et al. 2019 [36] | TRA  FLY | n: TRA = 16 (8 female)  FLY = 16 (8 female)  Age = 26.0 ± 4.0  Population: recreationally active | 8 weeks  2-3 sessions/week | FLY: inertial load = 0.075 kg^.^m^2^ (males) & 0.05 kg^.^m^2^ (females) | Strength: 1RM knee extension  Power: Flywheel knee extension peak power  Hypertrophy: Quadriceps CSA (MRI) |
| MacDonald et al. 2012 [37] | TRA  PTC | n: TRA = 11 (0 female)  PTC = 10 (0 female)  Age = 21.73 ± 3.40  Population: recreationally trained | 6 weeks  2 sessions/week | PTC: Complex pairs (high-bar back squat + lateral jumps; Romanian deadlift)  Intra-pair rest = 3 minutes | Strength: 1RM back squat |
| Mao et al. 2023 [38] | TRA  RRS | n: TRA = 15 (0 female)  RRS = 15 (0 female)  Age: TRA = 23.9 ± 3.9  RRS = 22.7 ± 1.9  Population: active college students | 8 weeks  2 sessions/week | RRS: RRS rest = 51 seconds  RRS frequency = 8 | Strength: 1RM back squat  Power: Back squat mean power  Hypertrophy: Rectus femoris muscle thickness (ultrasound) |
| Maroto-Izquierdo et al. 2017 [39] | TRA  FLY | n: TRA = 15 (0 female)  FLY = 14 (0 female)  Age: TRA = 23.8 ± 1.6  FLY = 19.8 ± 1.0  Population: handball players | 6 weeks  2-3 sessions/week | FLY: inertial load = 0.145 kg^.^m^2^ | Strength: 1RM leg press  Power: Leg press concentric power (50% 1RM)  Jump: CMJ height  Hypertrophy: Vastus lateralis muscle thickness (ultrasound)  Sprint: 20-metre time |
| McCurdy et al. 2009 [40] | TRA  ACC | n: TRA = 14  FLY = 14  Age = 20.63 ± 1.33  Population: baseball players | 9 weeks  2 sessions/week | ACC: Load of plates in TRA replaced with chains for ACC | Strength: 1RM bench press |
| Melo et al. 2013 [41] | TRA  ACC | n: TRA = 15 (6 female)  FLY = 14 (5 female)  Age: TRA = 23.8 ± 1.6  FLY = 19.8 ± 1.0  Population: physically active | 12 weeks  3 sessions/week | ACC: Elastic bands (~3-7kg at end of range of motion) | Strength: 1RM knee extension |
| Merrigan et al. 2019 [42] | TRA  SUP | n: TRA = 12 (12 female)  SUP = 10 (10 female)  Age: TRA = 20.9 ± 3.4  SUP = 20.6 ± 1.1  Population: recreationally active | 12 weeks  2 sessions/week | SUP: superset pairs (smith machine squat + leg press) | Strength: 1RM squat  Hypertrophy: Vastus lateralis CSA (ultrasound) |
| Naczk et al. 2024 [43] | TRA  FLY | n: TRA = 13 (0 female) FLY = 13 (0 female)  Age = 20.40 ± 1.18  Population: recreationally active | 6 weeks  3 sessions/week | FLY: inertial load ~= 70% 1RM | Strength: 1RM knee extension  Jump: CMJ height |
| Nicholson et al. 2016 [44] | TRA  RRS | n: TRA = 11 (0 female)  RRS = 12 (0 female)  Age = 21.76 ± 2.6  Population: recreationally trained | 6 weeks  2 sessions/week | RRS: Cluster rest = 25 seconds  Cluster frequency = 6 | Strength: 1RM back squat  Power: 20kg jump squat power  Jump: CMJ height |
| Oliver et al. 2013 [45] | TRA  RRS | n: TRA = 11 (0 female)  RRS = 11 (0 female)  Age: TRA = 25.0 ± 5.0  RRS = 25.0 ± 4.0  Population: military operations and recreationally trained | 12 weeks  4 sessions/week | RRS: RRS rest = 60 seconds  RRS frequency = 2 | Strength: 1RM back squat  Power: Back squat power |
| Ozaki et al. 2018 [46] | TRA  DRO | n: TRA = 9 (0 female)  DRO = 9 (0 female)  Age = 26.0 ± 1.0  Population: untrained | 8 weeks  2-3 sessions/week | DRO: 20% 1RM reduction, 3 x 10% 1RM reductions | Strength: 1RM dumbbell curl  Hypertrophy: Elbow flexor CSA (MRI) |
| Pan et al. 2025 [47] | TRA  ACC | n: TRA = 15 (0 female)  ACC = 15 (0 female)  Age = 21.65 ± 2.2  Population: college students | 6 weeks  2 sessions/week | ACC: Elastic bands (20% 1RM of total load) | Strength: 1RM squat  Hypertrophy: Rectus femoris CSA (ultrasound)  Jump: CMJ height  Sprint: 20-metre time |
| Rathi et al. 2023 [48] | TRA PTC | n: TRA = 10 (10 female)  PTC = 11 (11 female)  Age: TRA = 21.1 ± 2.7  PTC = 20.0 ± 2.3  Population: team-sport athletes | 6 weeks  2 sessions/week | PTC: Contrast pairs (squat + squat jump; dumbbell lunge + split jump; bench press + push-up; Romanian deadlift + kettlebell swing)  Intra-pair rest = 3 minutes | Jump: Standing long jump distance  Sprint: 30-metre time |
| Sagelv et al. 2020 [49] | TRA  FLY | n: TRA = 13 (0 female)  FLY = 13 (0 female)  Age: TRA = 23.23 ± 2.12  FLY = 23.07 ± 3.15  Population: football players | 6 weeks  2 sessions/week | FLY: inertial load = 0.025 – 0.100 kg^.^m^2^ | Strength: 1RM partial squat  Jump: CMJ height  Sprint: 10-metre time |
| Samson et al. 2018 [50] | TRA  RRS | n: TRA = 16 (0 female)  RRS = 16 (0 female)  Age = 22  Population: recreationally active | 7 weeks  3 sessions/week | RRS: RRS rest = 15 seconds  RRS frequency = 8 | Strength: 1RM back squat |
| Schneiker et al. 2023 [51] | TRA  PTC | n: TRA = 8 (0 female)  PTC = 8 (0 female)  Age = 19.0 ± 2.0  Population: sub-elite Australian rules football players | 6 weeks  4 sessions/week | PTC: Complex pairs (squat + squat jump; deadlift + clean pulls)  Intra-pair rest = 3 minutes | Strength: 1RM half-squat  Power: 30% 1RM jump squat peak power  Jump: Vertical jump  Sprint: 20-meter time |
| Shoepe et al. 2011 [52] | TRA  ACC | n: TRA = 10 (5 female)  ACC = 10 (5 female)  Age: TRA = 19.9 ± 1.2  ACC = 20.0 ± 1.4  Population: university students | 24 weeks  3 sessions/week | ACC: Elastic bands (20-35% 1RM of total load) | Strength: 1RM squat  Power: 90-degree isokinetic knee extension power |
| Stasinaki et al. 2015 [53] | TRA  PTC | n: TRA = 9 (0 female)  PTC = 9 (0 female)  Age: TRA = 22.3 ± 2.7  PTC = 21.9 ± 2.3  Population: moderately trained physical education students | 6 weeks  3 sessions/week | PTC: Complex pairs (leg press + leg press throw; bench press + bench press throw; smith box squat + squat jump)  Intra-pair rest = 5 minutes | Strength: 1RM box squat  Jump: CMJ height  Hypertrophy: Vastus lateralis muscle thickness (ultrasound) |
| Talpey et al. 2016 [54] | TRA  PTC | n: TRA = 11 (0 female)  PTC = 9 (0 female)  Age: TRA = 20.91 ± 3.59  PTC = 21.44 ± 3.54  Population: recreationally trained | 9 weeks  2 sessions/week | PTC: Contrast pairs (half squat + squat jump)  Intra-pair rest = 4 minutes | Strength: 1RM half squat  Jump: CMJ height  Power: CMJ power  Sprint: 20-metre time |
| Tøien et al. 2018 [55] | TRA  AEL | n: TRA = 19 (0 female)  AEL = 19 (0 female)  Age: TRA = 24.0 ± 2.0  AEL = 22.0 ± 3.0  Population: healthy non-smoking | 8 weeks  3 sessions/week | AEL: additional eccentric load = 55-60% 1RM | Strength: 1RM leg press  Jump: CMJ height |
| Vargas-Molina et al. 2025 [56] | TRA  RRS | n: TRA = 10 (3 female)  RRS = 10 (3 female)  Age = 21.5 ± 1.5  Population: resistance trained adults | 8 weeks  2 sessions/week | RRS: Cluster rest = 20 seconds  Cluster frequency = 3 | Hypertrophy: Rectus femoris muscle thickness (ultrasound) |
| Varovic et al. 2021 [57] | TRA  DRO | n: TRA = 16 (0 female)  AEL = 16 (0 female)  Age = 19.21 ± 1.10  Population: physically active | 8 weeks  1-3 sessions/week | DRO: 20% 1RM reduction, 10-15% 1RM reduction | Strength: Isokinetic knee extension peak torque  Hypertrophy: Vastus lateralis muscle thickness (ultrasound) |
| Walker et al. 2016 [58] | TRA  AEL | n: TRA = 10 (0 female)  AEL = 10 (0 female)  Age: TRA = 24.0 ± 4.0  AEL = 21.0 ± 2.0  Population: recreationally trained | 10 weeks  2 sessions/week | AEL: additional eccentric load = 40% 1RM | Strength: 1RM unilateral knee extension  Hypertrophy: Vastus lateralis CSA (ultrasound) |
| Weng et al. 2022 [59] | TRA  FLY | n: TRA = 10 (0 female)  FLY = 12 (0 female)  Age: TRA = 21.4 ± 2.5  FLY = 22.2 ± 2.6  Population: long-distance runners | 6 weeks  3 sessions/week | FLY: inertial load = 0.06 kg^.^m^2^ | Jump: CMJ height |
| Xie et al. 2024 [60] | TRA  FLY | n: TRA = 10 (10 female)  FLY (10 female)  Age: TRA = 20.2 ± 2.6  FLY = 20.7 ± 2.0  Population: college basketball players | 6 weeks  2 sessions/week | FLY: inertial load = 0.075 kg^.^m^2^ | Strength: 1RM squat  Jump: CMJ height |
| Yuan et al. 2025 [61] | TRA  FLY | n: TRA = 9 (0 female)  FLY = 8 (0 female)  Age: TRA = 21.14 ± 2.12  FLY = 21.57 ± 2.23  Population: elite badminton players | 6 weeks  2 sessions/week | FLY: inertial load = Pro + Large plate – Pro + Medium + Small plate | Strength: Isometric mid-thigh pull peak force |
| Zhu et al. 2024 [62] | TRA  RRS | n: TRA = 8 (0 female)  RRS = 8 (0 female)  Age: TRA = 19.6 ± 1.1  FLY = 19.5 ± 1.1  Population: table tennis players | 8 weeks  3 sessions/week | RRS: Cluster rest = 30 seconds  Cluster frequency = 1 | Strength: 1RM bench press  Power: Wingate peak power |

*Abbreviations:* ACC, accommodating resistance; AEL, accentuated eccentric loading; DRO, dropsets; FLY, flywheel; PTC, potentiation complexes; RRS, rest redistribution schemes; SUP, supersets; TRA, traditional resistance training; CMJ, countermovement jump; 1RM, one-repetition maximum; MVIT, maximal voluntary isometric torque; CSA, cross-sectional area; MRI, magnetic resonance imaging; rad^.^s, radian per second; kg, kilogram; OPL, optimum power load; kg^.^m^2^, kilograms per meter squared.

1.	Alemdaroğlu U, Dündar U, Köklü Y, Aşci A, Findikoğlu G. The effect of exercise order incorporating plyometric and resistance training on isokinetic leg strength and vertical jump performance: A comparative study. Isokinetics & Exercise Science. 2013;21(3):211-7. <https://doi.org/10.3233/IES-130509>

2.	Andersen V, Fimland MS, Kolnes MK, Saeterbakken AH. Elastic Bands in Combination With Free Weights in Strength Training: Neuromuscular Effects. Journal of strength and conditioning research. 2015;29(10):2932-40. <https://doi.org/10.1519/JSC.0000000000000950>

3.	Angleri V, Ugrinowitsch C, Libardi C, Libardi CA. Crescent pyramid and drop-set systems do not promote greater strength gains, muscle hypertrophy, and changes on muscle architecture compared with traditional resistance training in well-trained men. European Journal of Applied Physiology. 2017;117(2):359-69. <https://doi.org/10.1007/s00421-016-3529-1>

4.	Arazi H, Asadi A, Roohi S. Enhancing muscular performance in women: compound versus complex, traditional resistance and plyometric training alone. J Musculoskelet Res. 2014;17(02):1450007. <https://doi.org/https://doi.org/10.1142/S0218957714500079>

5.	Arazi H, Khanmohammadi A, Asadi A, Haff GG. The effect of resistance training set configuration on strength, power, and hormonal adaptation in female volleyball players. Applied Physiology, Nutrition & Metabolism. 2018;43(2):154-64. <https://doi.org/10.1139/apnm-2017-0327>

6.	Arazi H, Salek L, Nikfal E, Izadi M, Tufano JJ, Elliott BT, et al. Comparable endocrine and neuromuscular adaptations to variable vs. constant gravity-dependent resistance training among young women. Journal of translational medicine. 2020;18(1):239. <https://doi.org/10.1186/s12967-020-02411-y>

7.	Arazi H, Khoshnoud A, Asadi A, Tufano JJ. The effect of resistance training set configuration on strength and muscular performance adaptations in male powerlifters. Scientific reports. 2021;11(1):7844. <https://doi.org/10.1038/s41598-021-87372-y>

8.	Arsenis S, Gioftsidou A, Smilios I, Malliou P, Chatzinikolaou A. The effect of periodized flywheel training on power of lower limbs. The Journal of sports medicine and physical fitness. 2021;61(12):1563-9. <https://doi.org/10.23736/S0022-4707.20.11940-6>

9.	Banks NF, Berry AC, Rogers EM, Jenkins ND. Flywheel resistance training promotes unique muscle architectural and performance‐related adaptations in young adults. Eur J Sport Sci. 2024;24(12):1765-78. <https://doi.org/https://doi.org/10.1002/ejsc.12215>

10.	Baruah J, Autade SS, Thapa RK. Effects of complex-contrast versus traditional resistance training on linear sprint, change-of-direction speed, jumps, and maximal strength of physically active males. Int J Kinesiol Sports Sci. 2024;12(2):1. <https://doi.org/http://dx.doi.org/10.7575/aiac.ijkss.v.12n.2p.1>

11.	Brandenburg JP, Docherty D. The effects of accentuated eccentric loading on strength, muscle hypertrophy, and neural adaptations in trained individuals. J Strength Cond Res. 2002;16(1):25-32.

12.	Brito J, Vasconcellos F, Oliveira J, Krustrup P, Rebelo A. Short-term performance effects of three different low-volume strength-training programmes in college male soccer players. J Hum Kinet. 2014;40:121. <https://doi.org/https://doi.org/10.2478/hukin-2014-0014>

13.	Burnham TR, Ruud JD, McGowan R. Bench Press Training Program with Attached Chains for Female Volleyball and Basketball Athletes. Percept and Mot Skills. 2010 2010/02/01;110(1):61-8. <https://doi.org/https://doi.org/10.2466/pms.110.1.61-68>

14.	Caruso JF, Coday MA, Ramsey CA, Griswold SH, Polanski DW, Drummond JL, et al. Leg and Calf Press Training Modes and Their Impact on Jump Performance Adaptations. J Strength Cond Res. 2008;22(3):766-72. <https://doi.org/10.1519/JSC.0b013e31816a849a>

15.	Cin M, Çabuk R, Demirarar O, Özçaldiran B. Cluster resistance training results higher improvements on sprint, agility, strength and vertical jump in professional volleyball players. Turkiye Klin J Sports Sci. 2021;13(2):234-40. <https://doi.org/10.5336/sportsci.2020-79052>

16.	Cornejo-Daza PJ, Sánchez-Valdepeñas J, Rodiles-Guerrero L, Boullosa D, León-Prados JA, Wernbom M, et al. Training Effects of Traditional versus Cluster Set Configuration with and without Blood Flow Restriction. Med Sci Sports and Exerc. 2024;57(4):668-79. <https://doi.org/https://doi.org/10.1249/mss.0000000000003600>

17.	Cuevas-Aburto J, Jukic I, González-Hernández JM, Janicijevic D, Barboza-González P, Chirosa-Ríos LJ, et al. Effect of Resistance-Training Programs Differing in Set Configuration on Maximal Strength and Explosive-Action Performance. Int J Sports Physiol Perform. 2021;16(2):243-9. <https://doi.org/https://doi.org/10.1123/ijspp.2019-1005>

18.	Dai J, Liang X, Wang H, Lin W, Sun J, Li D, et al. The Effect of Elastic Band Variable Resistance Training on the Explosive Power of Lower Limbs in College Basketball Athletes. J Strength Cond Res. 2025;39(8):e1003-e9. <https://doi.org/10.1519/JSC.0000000000004988>

19.	Djuric S, Cuk I, Sreckovic S, Mirkov D, Nedeljkovic A, Jaric S. Selective Effects of Training Against Weight and Inertia on Muscle Mechanical Properties. International Journal of Sports Physiology & Performance. 2016;11(7):927-32. <https://doi.org/10.1123/ijspp.2015-0527>

20.	Douglas J, Pearson S, Ross A, McGuigan M. Effects of Accentuated Eccentric Loading on Muscle Properties, Strength, Power, and Speed in Resistance-Trained Rugby Players. Journal of strength and conditioning research. 2018;32(10):2750-61. <https://doi.org/10.1519/JSC.0000000000002772>

21.	Eler S, Eler N. Cluster Method: Effects on Performance in Handball. International Journal of Disabilities Sports & Health Sciences. 2024;7(1):144-51. <https://doi.org/10.33438/ijdshs.1364845>

22.	Enes A, Alves RC, Schoenfeld BJ, Oneda G, Perin SC, Trindade TB, et al. Rest-pause and drop-set training elicit similar strength and hypertrophy adaptations compared with traditional sets in resistance-trained males. Applied physiology, nutrition, and metabolism = Physiologie appliquee, nutrition et metabolisme. 2021;46(11):1417-24. <https://doi.org/10.1139/apnm-2021-0278>

23.	Filetti C, Ruscello B, Leo I, Porta M, Chiari A, Miranda C, et al. Hypertrophic adaptations to a 6-week in-season barbell vs. flywheel squat added to regular soccer training. J Sports Med Phys Fit. 2022;63(1):60-8. <https://doi.org/https://doi.org/10.23736/s0022-4707.22.13793-x>

24.	Fink J, Schoenfeld BJ, Kikuchi N, Nakazato K. Effects of drop set resistance training on acute stress indicators and long-term muscle hypertrophy and strength. J Sports Med Phys Fitness. 2018 May;58(5):597-605. <https://doi.org/10.23736/s0022-4707.17.06838-4>

25.	Godard MP, Wygand JW, Carpinelli RN, Catalano S, Otto RM. Effects of accentuated eccentric resistance training on concentric knee extensor strength. J Strength Cond Res. 1998;12(1):26-9.

26.	Hansen KT, Cronin JB, Pickering SL, Newton MJ. Does cluster loading enhance lower body power development in preseason preparation of elite rugby union players? J Strength Cond Res. 2011;25(8):2118-26. <https://doi.org/10.1519/JSC.0b013e318220b6a3>

27.	Iversen VM, Eide VB, Unhjem BJ, Fimland MS. Efficacy of Supersets Versus Traditional Sets in Whole-Body Multiple-Joint Resistance Training: A Randomized Controlled Trial. J Strength Cond Res. 2024;38(8):1372-8. <https://doi.org/10.1519/JSC.0000000000004819>

28.	Janicijevic D, González-Hernández JM, Jiménez-Reyes P, Márquez G, García-Ramos A. Longitudinal effects of traditional and rest redistribution set configurations on explosive-strength and strength-endurance manifestations. J Strength Cond Res. 2023;37(5):980-6. <https://doi.org/10.1519/JSC.0000000000004376>

29.	Freitas TT, Calleja-González J, Carlos-Vivas J, Marín-Cascales E, Alcaraz PE. Short-term optimal load training vs a modified complex training in semi-professional basketball players. J Sports Sci. 2019 2019/02/16;37(4):434-42. <https://doi.org/https://doi.org/10.1080/02640414.2018.1504618>

30.	Friedmann-Bette B, Bauer T, Kinscherf R, Vorwald S, Klute K, Bischoff D, et al. Effects of strength training with eccentric overload on muscle adaptation in male athletes. Eur J Appl Physiol. 2010;108:821-36. <https://doi.org/https://doi.org/10.1007/s00421-009-1292-2>

31.	García-Orea GP, Rodríguez-Rosell D, Ballester-Sánchez Á, Da Silva-Grigoletto ME, Belando-Pedreño N. Upper-lower body super-sets vs. traditional sets for inducing chronic athletic performance improvements. PeerJ. 2023 2023/02/21;11:e14636. <https://doi.org/https://doi.org/10.7717/peerj.14636>

32.	Kashiani AB, Soh KG, Soh KL, Ong SL, Kittichottipanich B. Comparison between two methods of variable resistance training on body composition, muscular strength and functional capacity among untrained males. Malays J Mov Health Exerc. 2020;9(1):45-66.

33.	Li F, Wang R, Newton RU, Sutton D, Shi Y, Ding H. Effects of complex training versus heavy resistance training on neuromuscular adaptation, running economy and 5-km performance in well-trained distance runners. PeerJ. 2019 2019/04/25;7:e6787. <https://doi.org/https://doi.org/10.7717/peerj.6787>

34.	Li F, Nassis GP, Shi Y, Han G, Zhang X, Gao B, et al. Concurrent complex and endurance training for recreational marathon runners: Effects on neuromuscular and running performance. Eur J Sport Sci. 2021;21(9):1243-53. <https://doi.org/https://doi.org/10.1080/17461391.2020.1829080>

35.	Liu M, Zhou K, Li B, Guo Z, Chen Y, Miao G, et al. Effect of 12 weeks of complex training on occupational activities, strength, and power in professional firefighters. Front Physiol. 2022;13:962546. <https://doi.org/https://doi.org/10.3389/fphys.2022.962546>

36.	Lundberg TR, García-Gutiérrez MT, Mandić M, Lilja M, Fernandez-Gonzalo R. Regional and muscle-specific adaptations in knee extensor hypertrophy using flywheel versus conventional weight-stack resistance exercise. Applied physiology, nutrition, and metabolism = Physiologie appliquee, nutrition et metabolisme. 2019;44(8):827-33. <https://doi.org/10.1139/apnm-2018-0774>

37.	MacDonald CJ, Lamont HS, Garner JC. A Comparison of the Effects of 6 Weeks of Traditional Resistance Training, Plyometric Training, and Complex Training on Measures of Strength and Anthropometrics. Journal of strength and conditioning research. 2012;26(2):422-31. <https://doi.org/10.1519/JSC.0b013e318220df79>

38.	Mao J, Wang T, Wang Y, Kuan G. Effect of resistance training programs differing in set structure on muscular hypertrophy and performance in untrained young men. Frontiers in Physiology. 2023;14:1301535.

39.	Maroto-Izquierdo S, García-López D, de Paz JA. Functional and Muscle-Size Effects of Flywheel Resistance Training with Eccentric-Overload in Professional Handball Players. Journal of human kinetics. 2017;60:133-43. <https://doi.org/10.1515/hukin-2017-0096>

40.	McCurdy K, Langford G, Ernest J, Jenkerson D, Doscher M. Comparison of chain- and plate-loaded bench press training on strength, joint pain, and muscle soreness in Division II baseball players. J Strength Cond Res. 2009;23(1):187-95. <https://doi.org/10.1519/JSC.0b013e31818892b5>

41.	Melo MDO, Brodt GA, Gomes LE, Candotti CT, La Torre M, Loss JF. Effects of added elastic tubes on open-chain knee extensor strength training. Sports Biomech. 2013;12(2):195-203. <https://doi.org/https://doi.org/10.1080/14763141.2012.755706>

42.	Merrigan JJ, Jones MT, White JB. A Comparison of Compound Set and Traditional Set Resistance Training in Women: Changes in Muscle Strength, Endurance, Quantity, and Architecture. J Sci Sport Exerc. 2019 2019/11/01;1(3):264-72. <https://doi.org/https://doi.org/10.1007/s42978-019-00030-8>

43.	Naczk A, Kisiel-Sajewicz K, Gajewska E, Gramza P, Jędzrzejczak T, Naczk M. Is inertial training more effective than traditional resistance training in young healthy males? Front Physiol. 2024;15:1487624. <https://doi.org/https://doi.org/10.3389/fphys.2024.1487624>

44.	Nicholson G, Ispoglou T, Bissas A. The impact of repetition mechanics on the adaptations resulting from strength-, hypertrophy- and cluster-type resistance training. Eur J App Physiol. 2016;116(10):1875-88. <https://doi.org/https://doi.org/10.1007/s00421-016-3439-2>

45.	Oliver JM, Jagim AR, Sanchez AC, Mardock MA, Kelly KA, Meredith HJ, et al. Greater Gains in Strength and Power With Intraset Rest Intervals in Hypertrophic Training. The Journal of Strength & Conditioning Research. 2013;27(11).

46.	Ozaki H, Kubota A, Natsume T, Loenneke JP, Abe T, Machida S, et al. Effects of drop sets with resistance training on increases in muscle CSA, strength, and endurance: a pilot study. J Sports Sci. 2018 2018/03/19;36(6):691-6. <https://doi.org/https://doi.org/10.1080/02640414.2017.1331042>

47.	Pan C, Li W, Su Y, Shi Y. Effects of variable resistance training with different load ratios on lower-limb strength and power in male college students. J Exerc Sci Fit. 2025;23(3):181-9. <https://doi.org/https://doi.org/10.1016/j.jesf.2025.04.001>

48.	Rathi A, Sharma D, Thapa RK. Effects of complex-descending versus traditional resistance training on physical fitness abilities of female team sports athletes. Biomed Hum Kinet. 2023;15(1):148-58. <https://doi.org/doi:10.2478/bhk-2023-0018>

49.	Sagelv EH, Pedersen S, Nilsen LPR, Casolo A, Welde B, Randers MB, et al. Flywheel squats versus free weight high load squats for improving high velocity movements in football. A randomized controlled trial. BMC Sports Science, Medicine and Rehabilitation. 2020 2020/10/02;12(1):61. <https://doi.org/10.1186/s13102-020-00210-y>

50.	Samson A, Pillai PS. Effect of Cluster Training Versus Traditional Training on Muscular Strength among Recreationally Active Males- A Comparative Study. Indian J Physiother Occup Ther. 2018;12(1):122-7. <https://doi.org/10.5958/0973-5674.2018.00022.9>

51.	Schneiker KT, Fyfe JJ, Teo SY, Bishop DJ. Comparative effects of contrast training and progressive resistance training on strength and power-related measures in subelite Australian rules football players. J Strength Cond Res. 2023;37(7):1440-8. <https://doi.org/10.1519/JSC.0000000000004423>

52.	Shoepe TC, Ramirez DA, Rovetti RJ, Kohler DR, Almstedt HC. The Effects of 24 weeks of Resistance Training with Simultaneous Elastic and Free Weight Loading on Muscular Performance of Novice Lifters. Journal of Human Kinetics. 2011;29:93-106. <https://doi.org/10.2478/v10078-011-0043-8>

53.	Stasinaki A-N, Gloumis G, Spengos K, Blazevich AJ, Zaras N, Georgiadis G, et al. Muscle Strength, Power, and Morphologic Adaptations After 6 Weeks of Compound vs. Complex Training in Healthy Men. Journal of strength and conditioning research. 2015;29(9):2559-69. <https://doi.org/10.1519/JSC.0000000000000917>

54.	Talpey SW, Young WB, Saunders N. Is nine weeks of complex training effective for improving lower body strength, explosive muscle function, sprint and jumping performance? Int J Sports Sci Coach. 2016;11(5):736-45. <https://doi.org/https://doi.org/10.1177/1747954116667112>

55.	Tøien T, Pedersen Haglo H, Unhjem R, Hoff J, Wang E. Maximal strength training: the impact of eccentric overload. J Neurophysiol. 2018;120(6):2868-76. <https://doi.org/https://doi.org/10.1080/02640414.2024.2391657>

56.	Vargas-Molina S, García-Sillero M, Maroto-Izquierdo S, Baz-Valle E, Bautista-Mayorga B, Murri M, et al. Cluster sets and traditional sets elicit similar muscular hypertrophy: a volume and effort-matched study in resistance-trained individuals. Eur J App Physiol. 2025;125(6):1725-34. <https://doi.org/https://doi.org/10.1007/s00421-025-05712-6>

57.	Varović D, Žganjer K, Vuk S, Schoenfeld BJ. Drop-set training elicits differential increases in non-uniform hypertrophy of the quadriceps in leg extension exercise. Sports. 2021;9(9):119. <https://doi.org/https://doi.org/10.3390/sports9090119>

58.	Walker S, Blazevich AJ, Haff GG, Tufano JJ, Newton RU, Häkkinen K. Greater strength gains after training with accentuated eccentric than traditional isoinertial loads in already strength-trained men. Front Physiol. 2016;7:149. <https://doi.org/https://doi.org/10.3389/fphys.2016.00149>

59.	Weng Y, Liu H, Ruan T, Yang W, Wei H, Cui Y, et al. Effects of flywheel resistance training on the running economy of young male well-trained distance runners. Front Physiol. 2022;13:1060640. <https://doi.org/https://doi.org/10.3389/fphys.2022.1060640>

60.	Xie L, Qu W, Dai J, Xu J, Zhang W, Sun J, et al. The impact of flywheel resistance squat training on lower limb strength in female college basketball players. Front Physiol. 2024;15:1491957. <https://doi.org/https://doi.org/10.3389/fphys.2024.1491957>

61.	Yuan S, Lu Z, Tan S, Zhang Z, Jing S, Liu H, et al. Comparison of six-week flywheel and traditional resistance training on deceleration and dynamic balance in elite badminton players. Front Physiol. 2025;16:1491661. <https://doi.org/https://doi.org/10.3389/fphys.2025.1491661>

62.	Zhu X, Suo P, Liu F. Similar adaptive responses in the upper body physical performance of table tennis players following the traditional and cluster set resistance and plyometric training. Sci Rep. 2024;14(1):28001. <https://doi.org/https://doi.org/10.1038/s41598-024-78795-4>
