# Supplementary material for: The Effects of Advanced Resistance Training Prescription Methods on Strength, Power, Hypertrophy, and Performance Adaptations in Healthy Adults: A Systematic Review and Bayesian Network Meta-analysis
Source: Sports Med. 2026 Apr 8;56(8):1955–77. doi: 10.1007/s40279-026-02428-1 (PMC13457283; doi:10.1007/s40279-026-02428-1)
Supplement: Supplementary file 10 [file 40279_2026_2428_MOESM8_ESM.docx]

**Online Supplemental Material 6.** Relative effects between all resistance training conditions within the alternate analysis for each network.

| **STRENGTH** | PTC | AEL | ACC | TRA | FLY | RRS | DRO |
| --- | --- | --- | --- | --- | --- | --- | --- |
| PTC | **-** | -0.18  (-0.54 - 0.17) | -**0.30**  **(**-**0.60** - -**0.00)** | -**0.31**  **(**-**0.51** - -**0.11)** | -**0.33**  **(**-**0.64** - -**0.02)** | -**0.38**  **(**-**0.68** - -**0.10)** | -**0.46**  **(**-**0.83** - -**0.09)** |
| AEL | 0.18  (-0.17 - 0.54) | **-** | -0.12  (-0.48 - 0.25) | -0.13  (-0.42 - 0.16) | -0.14  (-0.52 - 0.23) | -0.20  (-0.56 - 0.16) | -0.27  (-0.70 - 0.15) |
| ACC | **0.30**  **(0.00** - **0.60)** | 0.12  (-0.25 - 0.48) | **-** | -0.01  (-0.23 - 0.22) | -0.03  (-0.35 - 0.30) | -0.08  (-0.39 - 0.23) | -0.16  (-0.54 - 0.23) |
| TRA | **0.31**  **(0.11** - **0.51)** | 0.13  (-0.16 - 0.42) | 0.01  (-0.22 - 0.23) | **-** | -0.02  (-0.25 - 0.22) | -0.07  (-0.29 - 0.14) | -0.15  (-0.46 - 0.16) |
| FLY | **0.33**  **(0.02** - **0.64)** | 0.14  (-0.23 - 0.52) | 0.03  (-0.30 - 0.35) | 0.02  (-0.22 - 0.25) | **-** | -0.06  (-0.37 - 0.26) | -0.13  (-0.52 - 0.26) |
| RRS | **0.38**  **(0.10** - **0.68)** | 0.20  (-0.16 - 0.56) | 0.08  (-0.23 - 0.39) | 0.07  (-0.14 - 0.29) | 0.06  (-0.26 - 0.37) | **-** | -0.07  (-0.45 - 0.30) |
| DRO | **0.46**  **(0.09** - **0.83)** | 0.27  (-0.15 - 0.70) | 0.16  (-0.23 - 0.54) | 0.15  (-0.16 - 0.46) | 0.13  (-0.26 - 0.52) | 0.07  (-0.30 - 0.45) | **-** |
| **POWER** | RRS | PTC | TRA |  |  |  |  |
| RRS | **-** | -0.05  (-0.54 - 0.42) | -0.22  (-0.63 - 0.17) | **-** | **-** | **-** | **-** |
| PTC | 0.05  (-0.42 - 0.54) | **-** | -0.17  (-0.43 - 0.09) | **-** | **-** | **-** | **-** |
| TRA | 0.22  (-0.17 - 0.63) | 0.17  (-0.09 - 0.43) | **-** | **-** | **-** | **-** | **-** |
| **JUMP** | FLY | RRS | PTC | TRA |  |  |  |
| FLY | **-** | -0.02  (-0.52 - 0.46) | -0.35  (-0.78 - 0.07) | -**0.36**  **(**-**0.68** - -**0.04)** | **-** | **-** | **-** |
| RRS | 0.02  (-0.46 - 0.52) | **-** | -0.33  (-0.80 - 0.13) | -0.34  (-0.71 - 0.04) | **-** | **-** | **-** |
| PTC | 0.35  (-0.07 - 0.78) | 0.33  (-0.13 - 0.80) | **-** | -0.01  (-0.28 - 0.27) | **-** | **-** | **-** |
| TRA | **0.36**  **(0.04** - **0.68)** | 0.34  (-0.04 - 0.71) | 0.01  (-0.27 - 0.28) | **-** | **-** | **-** | **-** |

***Note.*** Results for the strength network are from the sensitivity analysis, while results for the power and jump networks are from the initial analyses. Relative effects are presented as SMD (95% CrI), with a positive effect favouring the condition in the column header. Results in bold highlight a 95% probability that the favoured condition presents a greater relative effect in developing the respective physical capacity. *Abbreviations*: ACC, accommodating resistance; AEL, accentuated eccentric loading; DRO, dropsets; FLY, flywheel; PTC, potentiation complexes; RRS, rest redistribution schemes; TRA, traditional resistance training.
