# Supplementary material for: The Effects of Advanced Resistance Training Prescription Methods on Strength, Power, Hypertrophy, and Performance Adaptations in Healthy Adults: A Systematic Review and Bayesian Network Meta-analysis
Source: Sports Med. 2026 Apr 8;56(8):1955–77. doi: 10.1007/s40279-026-02428-1 (PMC13457283; doi:10.1007/s40279-026-02428-1)
Supplement: Supplementary file 11 [file 40279_2026_2428_MOESM9_ESM.docx]

**Online Supplemental Material 7:** Ranking Probability Percentages

**(a)**

**(c)**

**(e)**

**(b)**

**(d)**

**(f)**

**Initial Analysis**

**Sensitivity Analysis**

Probability of each condition’s ranking within the respective networks for strength (a), strength post-sensitivity analysis (b), power (c), power post-sensitivity analysis (d), jump performance (e), jump performance post-sensitivity analysis (f). *Abbreviations*: ACC, accommodating resistance; AEL, accentuated eccentric loading; DRO, dropsets; FLY, flywheel; PTC, potentiation complexes; RRS, rest redistribution schemes; TRA, traditional resistance training.

| **STRENGTH** | **Initial Analysis** | | | | | | |  | **Sensitivity Analysis** | | | | | | |
| --- | --- | --- | --- | --- | --- | --- | --- | --- | --- | --- | --- | --- | --- | --- | --- |
| **Rank** | **ACC** | **AEL** | **DRO** | **FLY** | **PTC** | **RRS** | **TRA** |  | **ACC** | **AEL** | **DRO** | **FLY** | **PTC** | **RRS** | **TRA** |
| 1 | 21.18 | 20.97 | 6.46 | 1.98 | 5.20 | 44.09 | 0.12 |  | 1.57 | 14.26 | 0.45 | 1.06 | 82.44 | 0.22 | 0.00 |
| 2 | 24.85 | 19.02 | 7.84 | 4.72 | 12.32 | 28.58 | 2.66 |  | 15.35 | 46.09 | 3.89 | 11.69 | 15.25 | 4.29 | 3.45 |
| 3 | 20.62 | 15.20 | 8.83 | 7.69 | 17.52 | 14.74 | 15.40 |  | 24.39 | 15.82 | 6.46 | 19.54 | 1.81 | 9.95 | 22.04 |
| 4 | 12.81 | 10.90 | 8.80 | 9.05 | 17.89 | 6.77 | 33.78 |  | 17.40 | 8.39 | 6.73 | 16.31 | 0.36 | 12.32 | 38.50 |
| 5 | 9.62 | 11.07 | 10.84 | 13.30 | 18.88 | 3.34 | 32.95 |  | 17.54 | 6.88 | 10.25 | 18.73 | 0.10 | 19.12 | 27.38 |
| 6 | 7.31 | 12.45 | 18.82 | 27.83 | 18.40 | 1.90 | 13.31 |  | 15.94 | 5.74 | 18.76 | 20.15 | 0.04 | 31.51 | 7.85 |
| 7 | 3.61 | 10.39 | 38.42 | 35.44 | 9.79 | 0.58 | 1.76 |  | 7.82 | 2.82 | 53.45 | 12.52 | 0.00 | 22.60 | 0.79 |
| **POWER** | **Initial Analysis** | | | | | | |  | **Sensitivity Analysis** | | | | | | |
| **Rank** | **PTC** | **RRS** | **TRA** |  |  |  |  |  | **PTC** | **RRS** | **TRA** |  |  |  |  |
| 1 | 59.00 | 39.71 | 1.29 |  |  |  |  |  | 37.52 | 56.08 | 6.40 |  |  |  |  |
| 2 | 28.90 | 52.59 | 18.51 |  |  |  |  |  | 24.70 | 32.29 | 43.01 |  |  |  |  |
| 3 | 12.10 | 7.70 | 80.20 |  |  |  |  |  | 37.79 | 11.62 | 50.59 |  |  |  |  |
| **JUMP** | **Initial Analysis** | | | | | | |  | **Sensitivity Analysis** | | | | | | |
| **Rank** | **FLY** | **PTC** | **RRS** | **TRA** |  |  |  |  | **FLY** | **PTC** | **RRS** | **TRA** |  |  |  |
| 1 | 53.21 | 1.34 | 45.40 | 0.04 |  |  |  |  | 85.66 | 0.84 | 12.88 | 0.62 |  |  |  |
| 2 | 42.25 | 9.18 | 46.26 | 2.30 |  |  |  |  | 12.43 | 9.93 | 50.21 | 27.43 |  |  |  |
| 3 | 3.68 | 43.45 | 5.74 | 47.12 |  |  |  |  | 1.42 | 21.19 | 18.75 | 58.65 |  |  |  |
| 4 | 0.85 | 46.02 | 2.60 | 50.53 |  |  |  |  | 0.50 | 68.04 | 18.15 | 13.30 |  |  |  |

*Abbreviations*: ACC, accommodating resistance; AEL, accentuated eccentric loading; DRO, dropsets; FLY, flywheel; PTC, potentiation complexes; RRS, rest redistribution schemes; TRA, traditional resistance training.
